# A taxonomic index, with names of descriptive authorities of termite genera and species: An accompaniment to *Biology of Termites: A Modern Synthesis* (Bignell DE, Roisin Y, Lo N, Editors. 2011. Springer, Dordrecht. 576 pp.)

**DOI:** 10.1093/jis/14.1.81

**Published:** 2014-06-22

**Authors:** D. E. Bignell, D. T. Jones

**Affiliations:** 1 Institute for Tropical Biology and Conservation, Universiti Malaysia Sabah, 88999 Kota Kinabalu, Sabah, Malaysia; 2 Present address: School of Biological and Chemical Sciences, Queen Mary, University of London, U.K. E1 4N; 3 Soil Biodiversity Group, Natural History Museum, London, UK SW7 5BD

**Keywords:** Biology of Termites 2011, taxonomic index, descriptive authorities

## Abstract

*Biology of Termites: A Modern Synthesis* (Bignell DE, Roisin Y, Lo N, (Editors), Springer, Dordrecht, 576pp, ISBN 978-90-481-3976-7, e-ISBN 978-90-481-3977-4, DOI 10.1007/978-90-481-3977-4) was published in 2011. With the agreement of the publishers, we give a taxonomic index of the book comprising 494 termite entries, 103 entries of other multicellular animal species mentioned as associates or predators of termites, with 9 fungal, 60 protist, and 64 prokaryote identities, which are listed as termite symbionts ( *sensu stricto* ). In addition, we add descriptive authorities for living (and some fossil) termite genera and species. Higher taxonomic groupings for termites are indicated by 25 code numbers. Microorganisms (prokaryotes, protists, and fungi) are listed separately, using broad modern taxonomic affiliations from the contemporary literature of bacteriology, protozoology, and mycology.

## Introduction

*Biology of Termites: A Modern Synthesis* (Bignell DE, Roisin Y, Lo N, (Editors), Springer, Dordrecht, 576pp, ISBN 978-90-481-3976-7, e-ISBN 978-90-481-3977-4, DOI 10.1007/978-90-481-3977-4) was published in 2011, a decade after *Termites: Evolution, Sociality, Symbioses, Ecology* (Abe T, Bignell DE, Higashi M (Editors), Kluwer Academic Publishers, Dordrecht, 466pp, ISBN 0-7923-6361-2), to which it was the intended successor, though with a different balance of topics. Both books lacked a taxonomic index, though an index to Abe et al. (2000) was eventually published ( Bignell and Jones 2009 ). In this paper we present a full taxonomic index for the 2011 book, encompassing termites, other animals associated with termites, and microbial symbionts ( *sensu stricto* ). For greater usefulness, we add descriptive authorities for termite genera and species and allocate each entry to one of 25 higher taxonomic groupings (or functional taxonomic group, FTG), following recent views of termite phylogeny and the genus lists presented in Chapter 17 of the book ( Jones and Eggleton 2011 ). Use of the term “clade” is avoided. Inevitably, compilation of the index has revealed a small number of taxonomic errors in the text of the book. The more serious of these are noted below.

## Materials and Methods

The termite index is presented as Table 1 . Entries are listed in six categories:

**Table 1:**
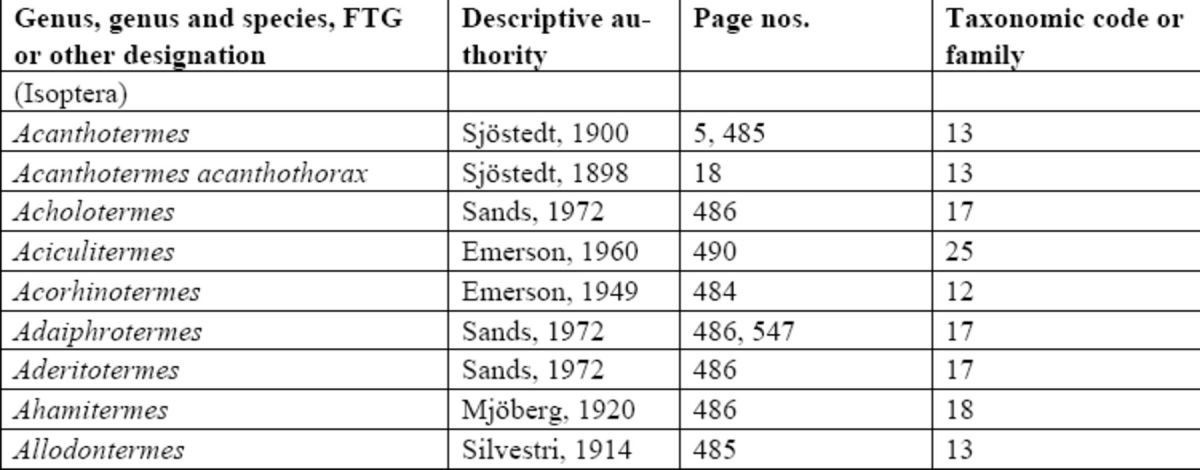

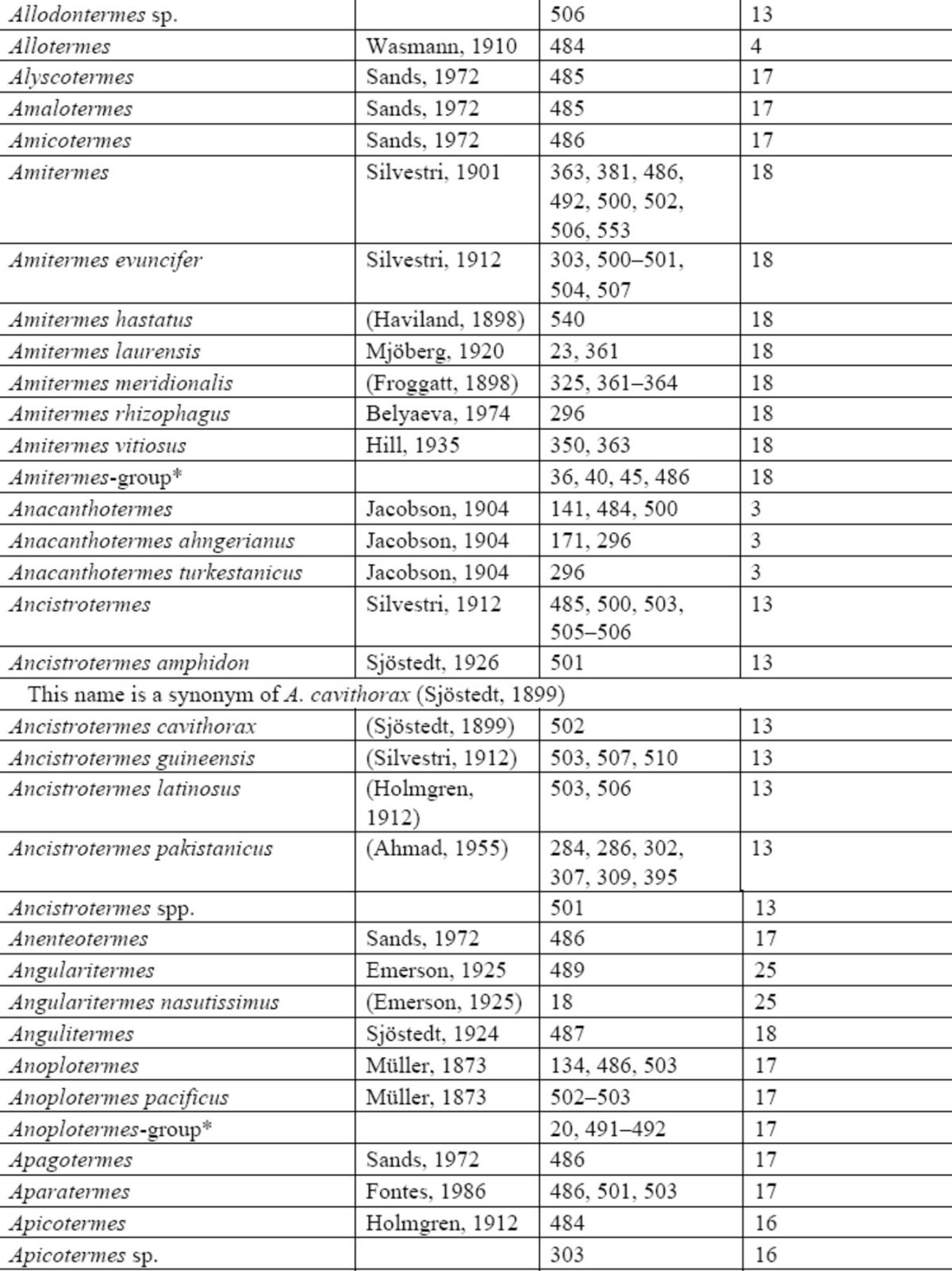

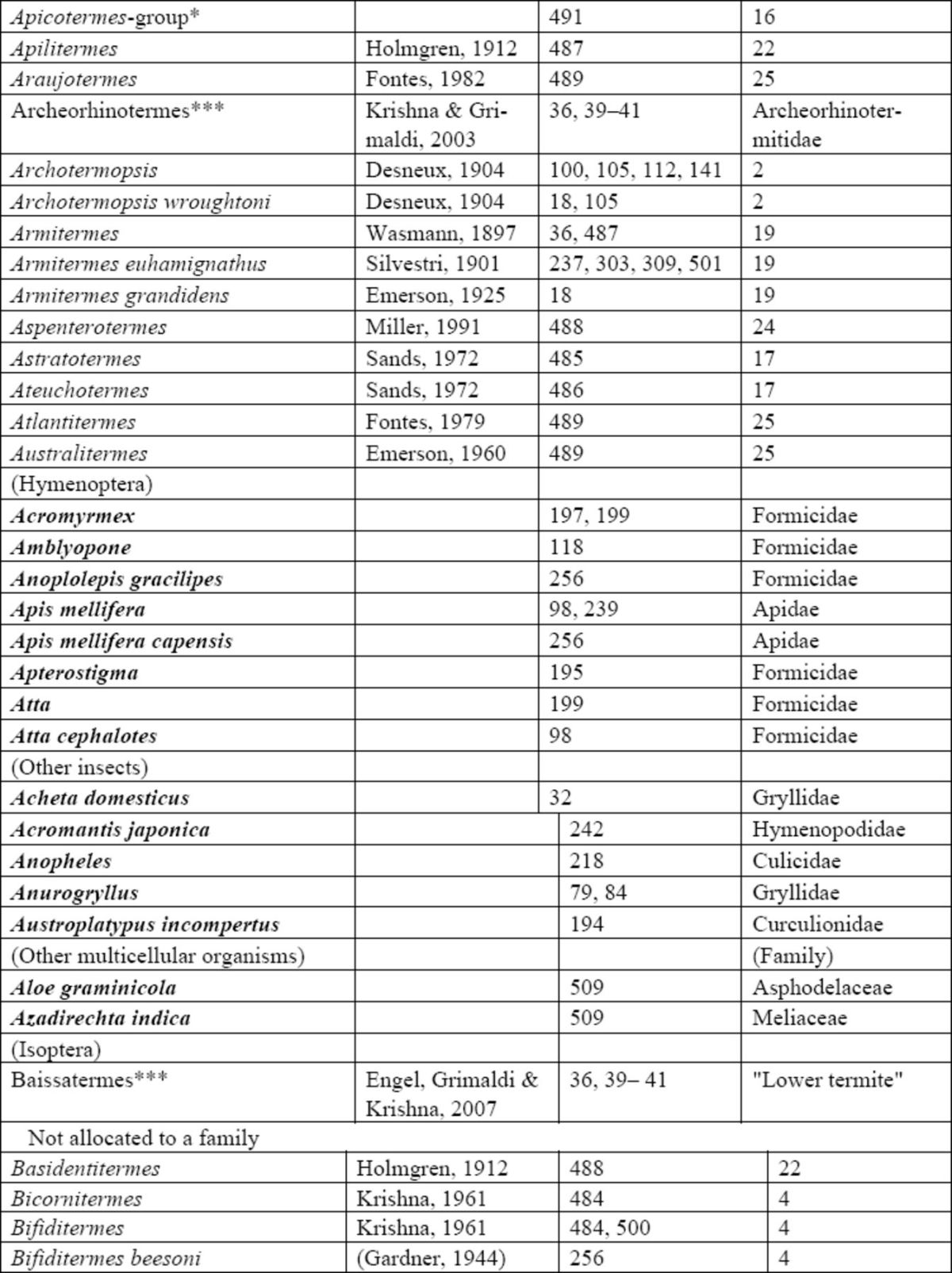

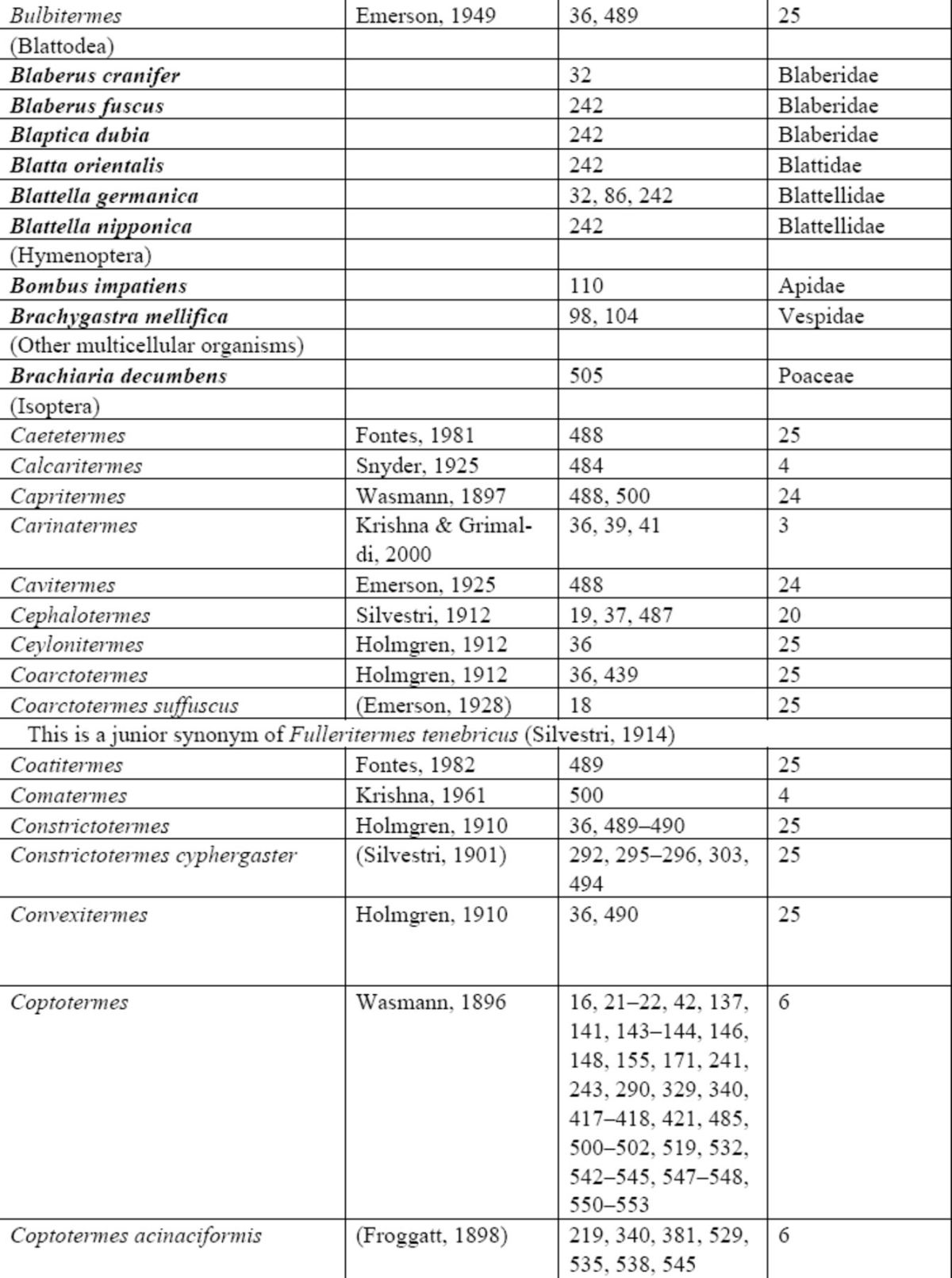

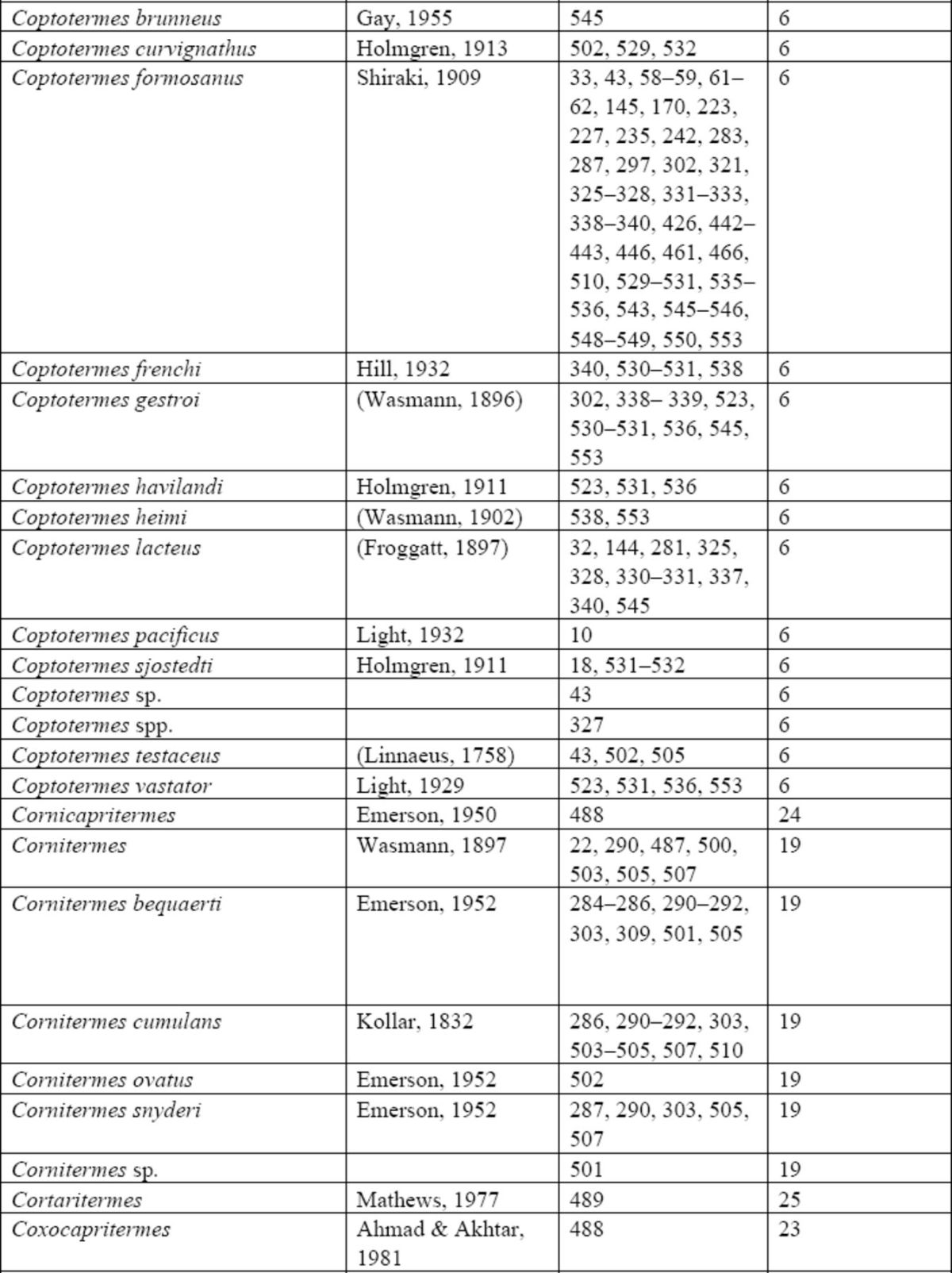

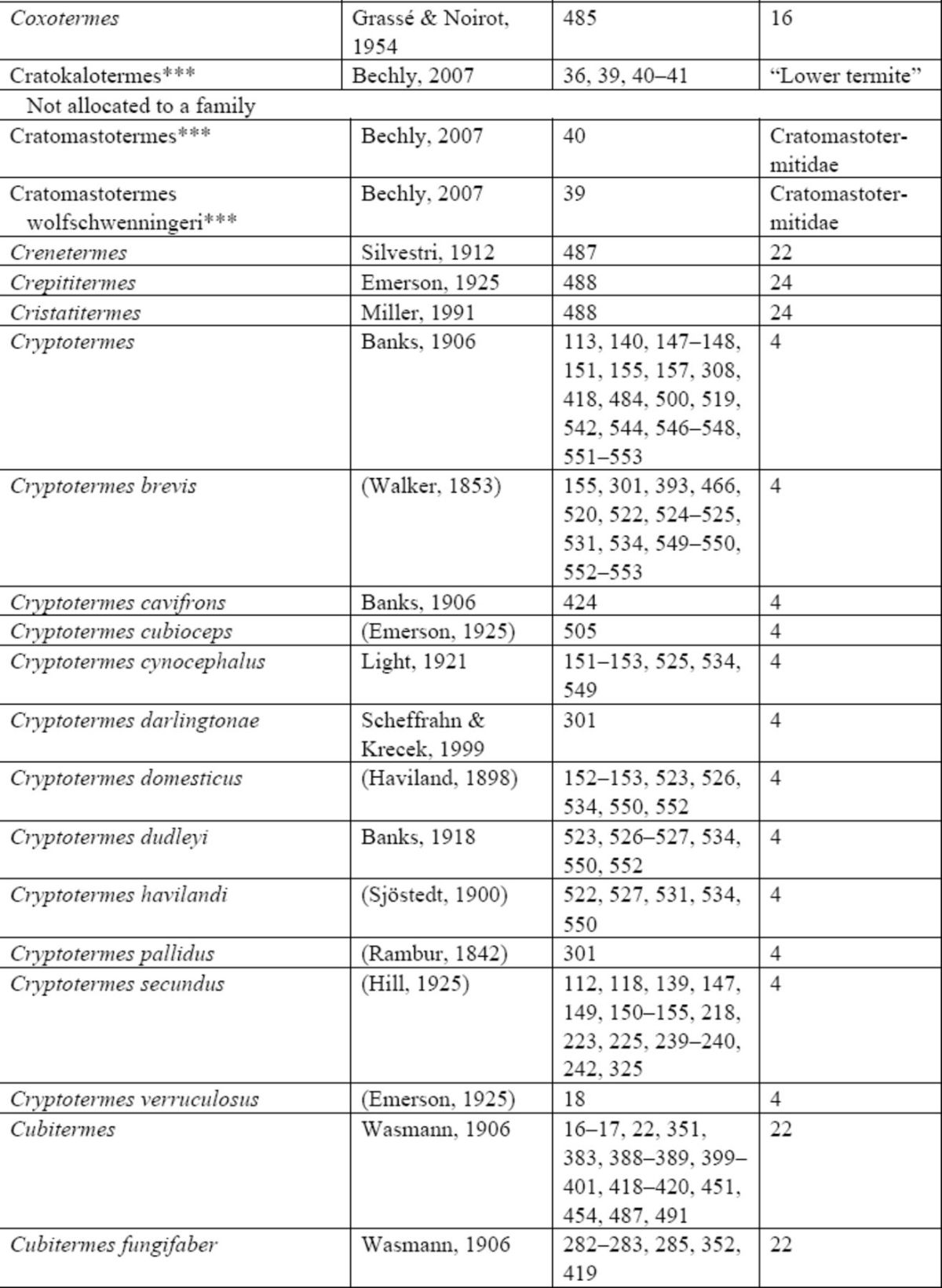

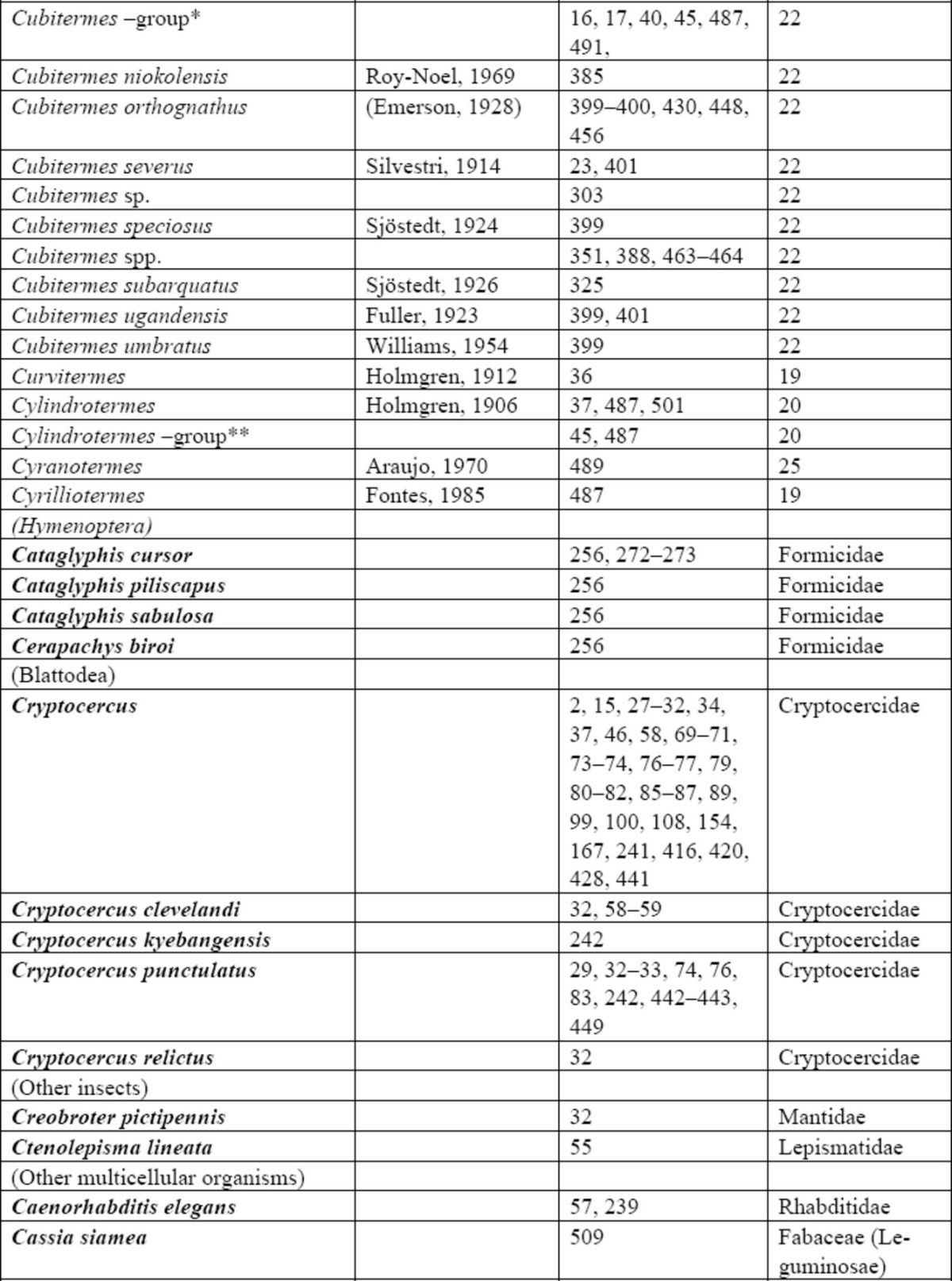

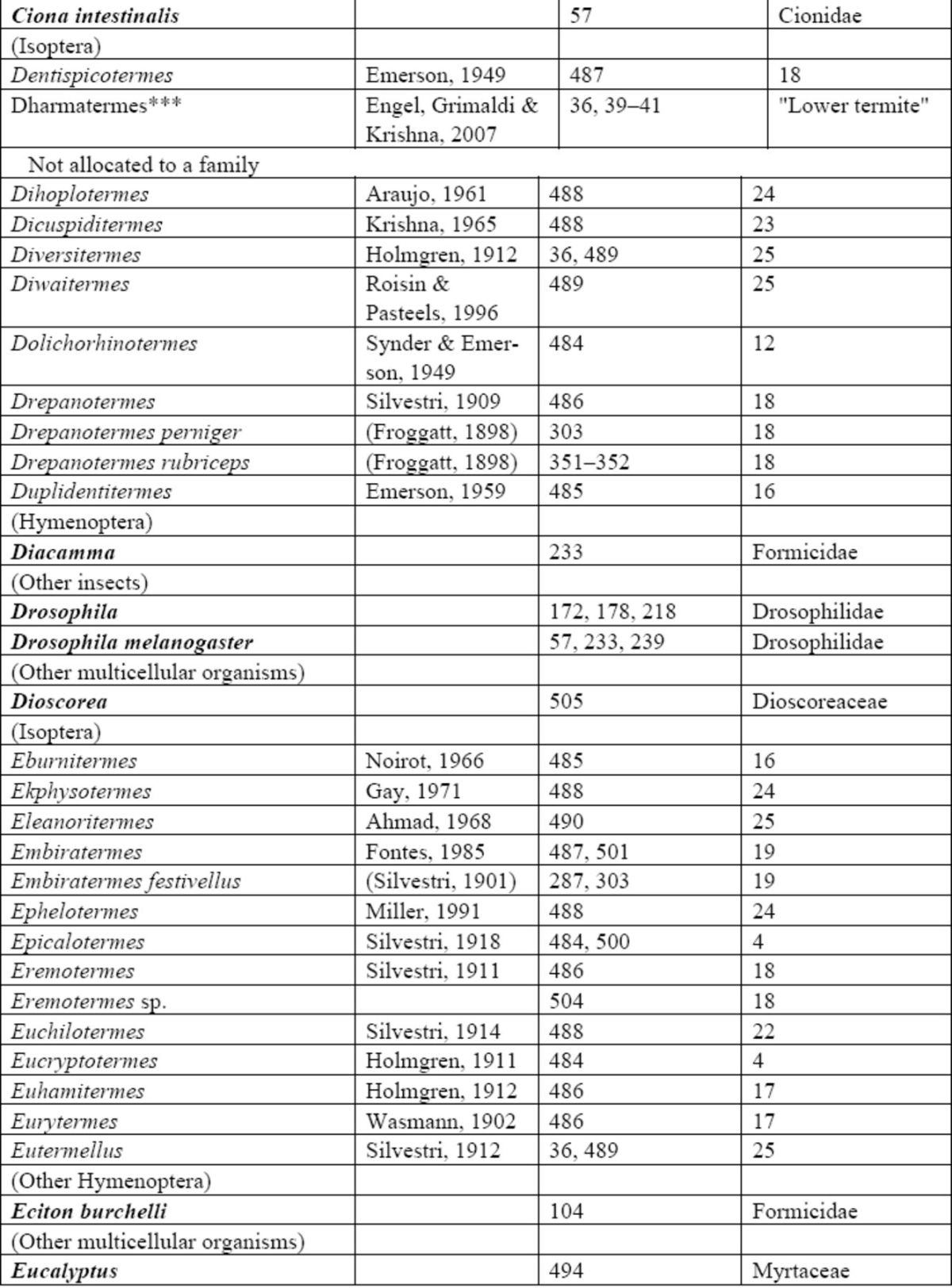

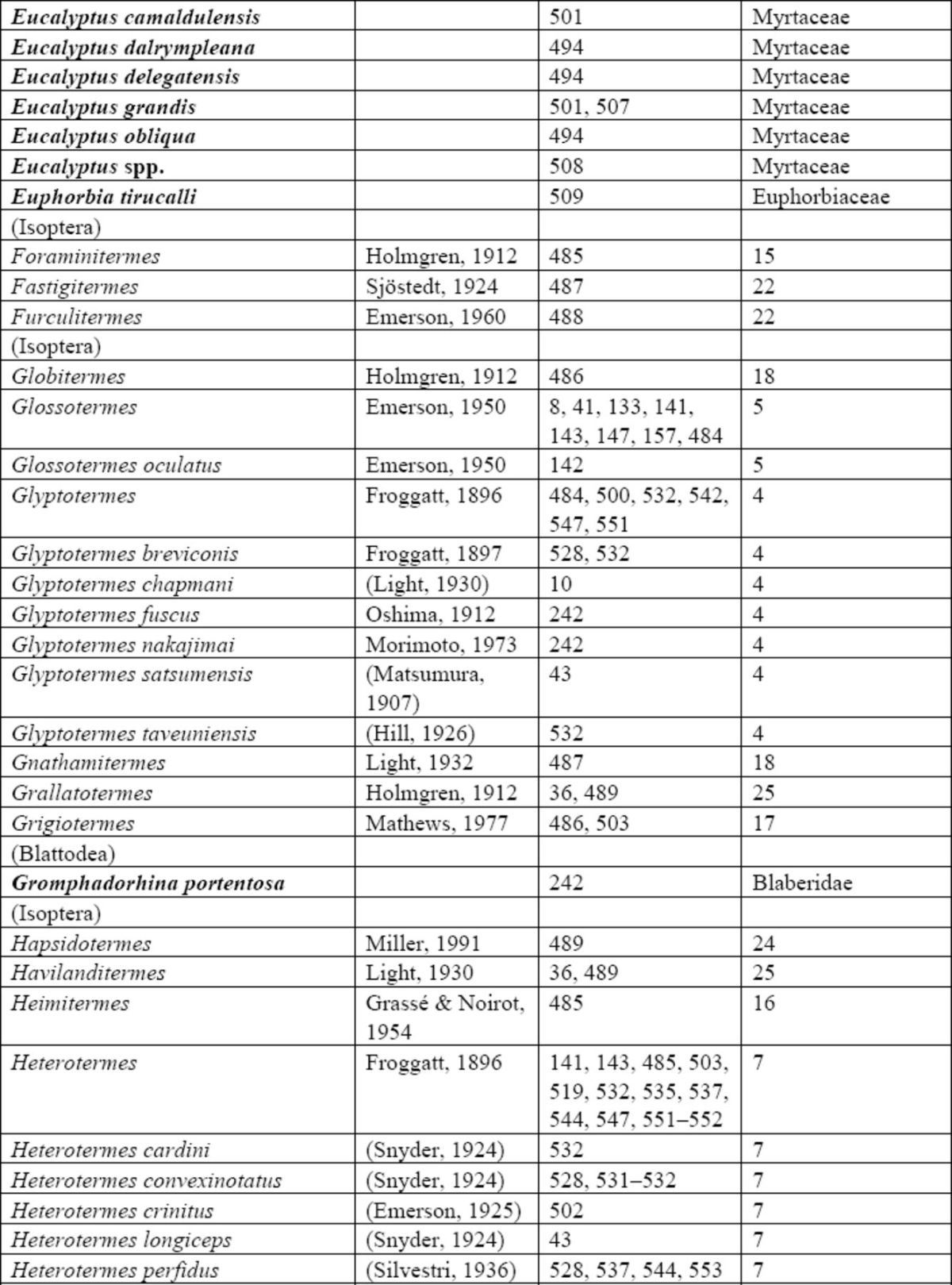

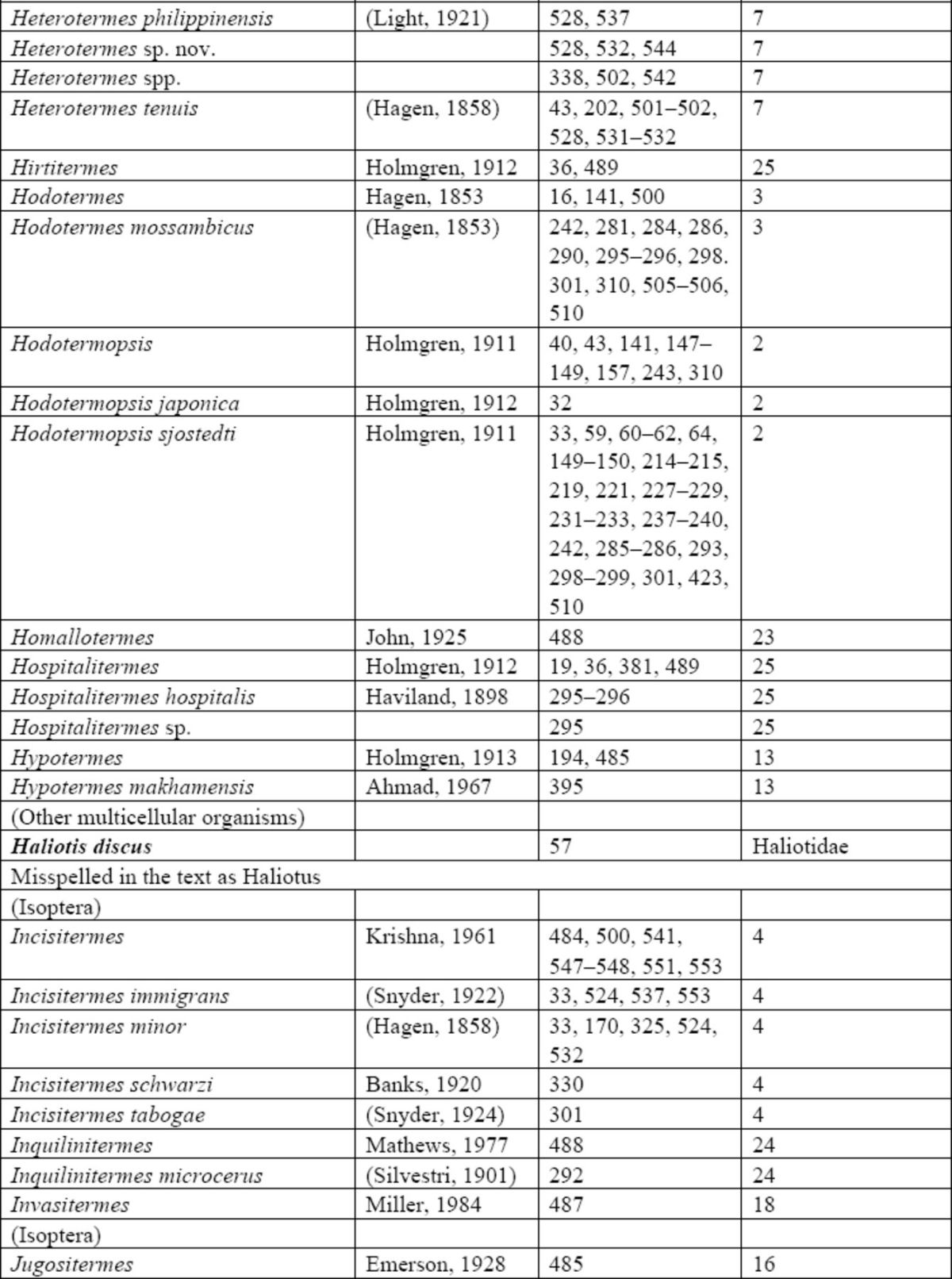

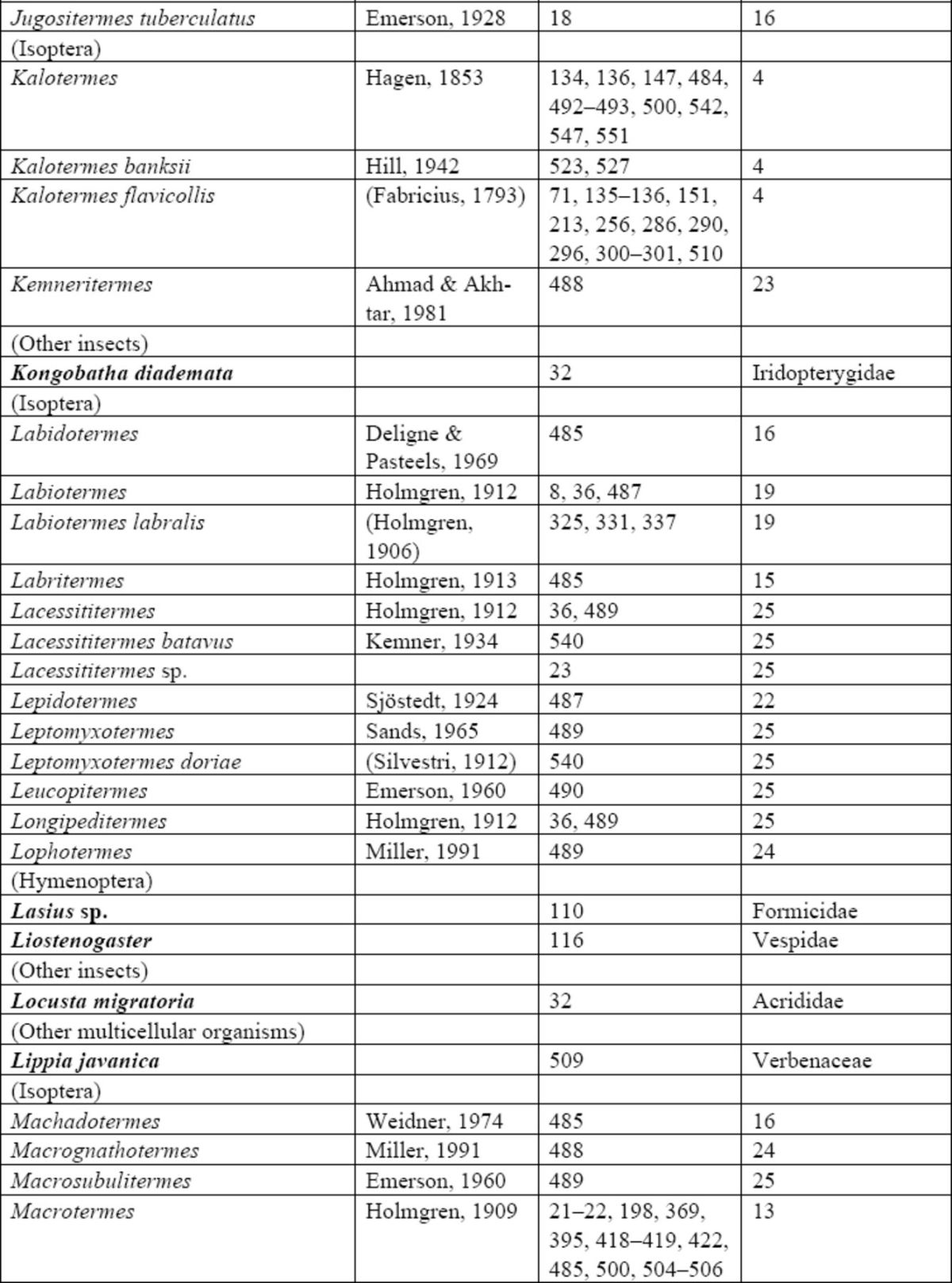

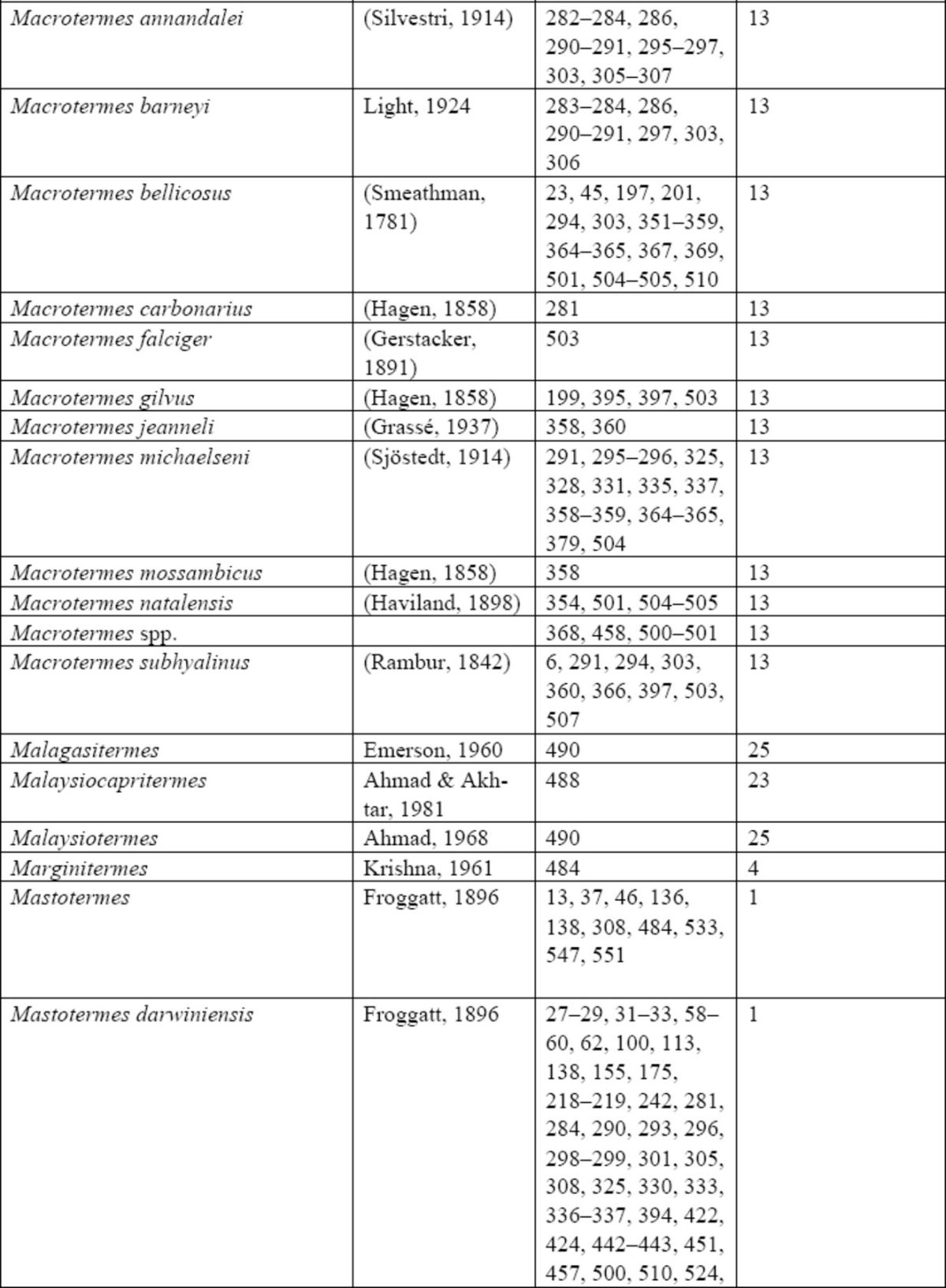

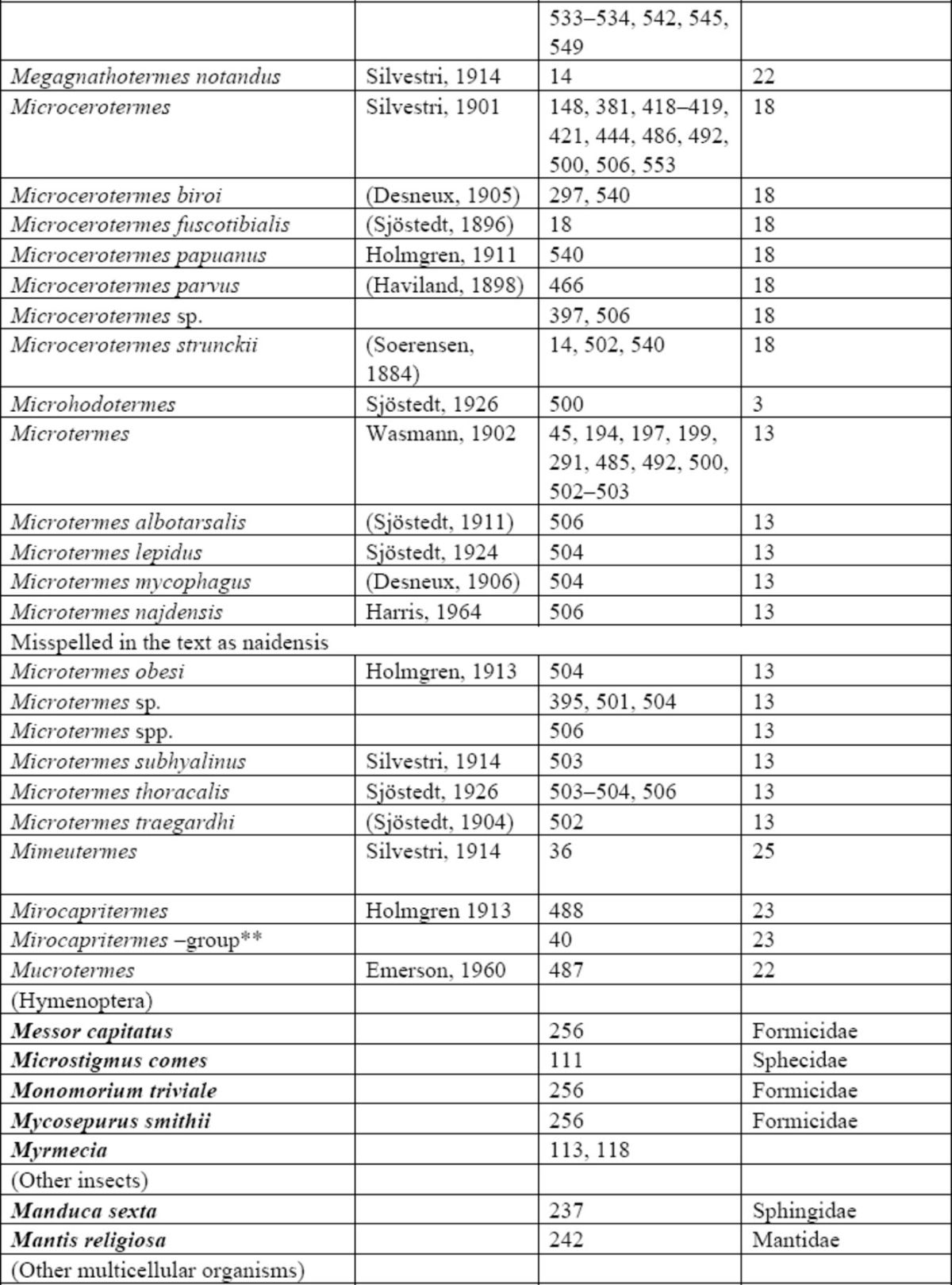

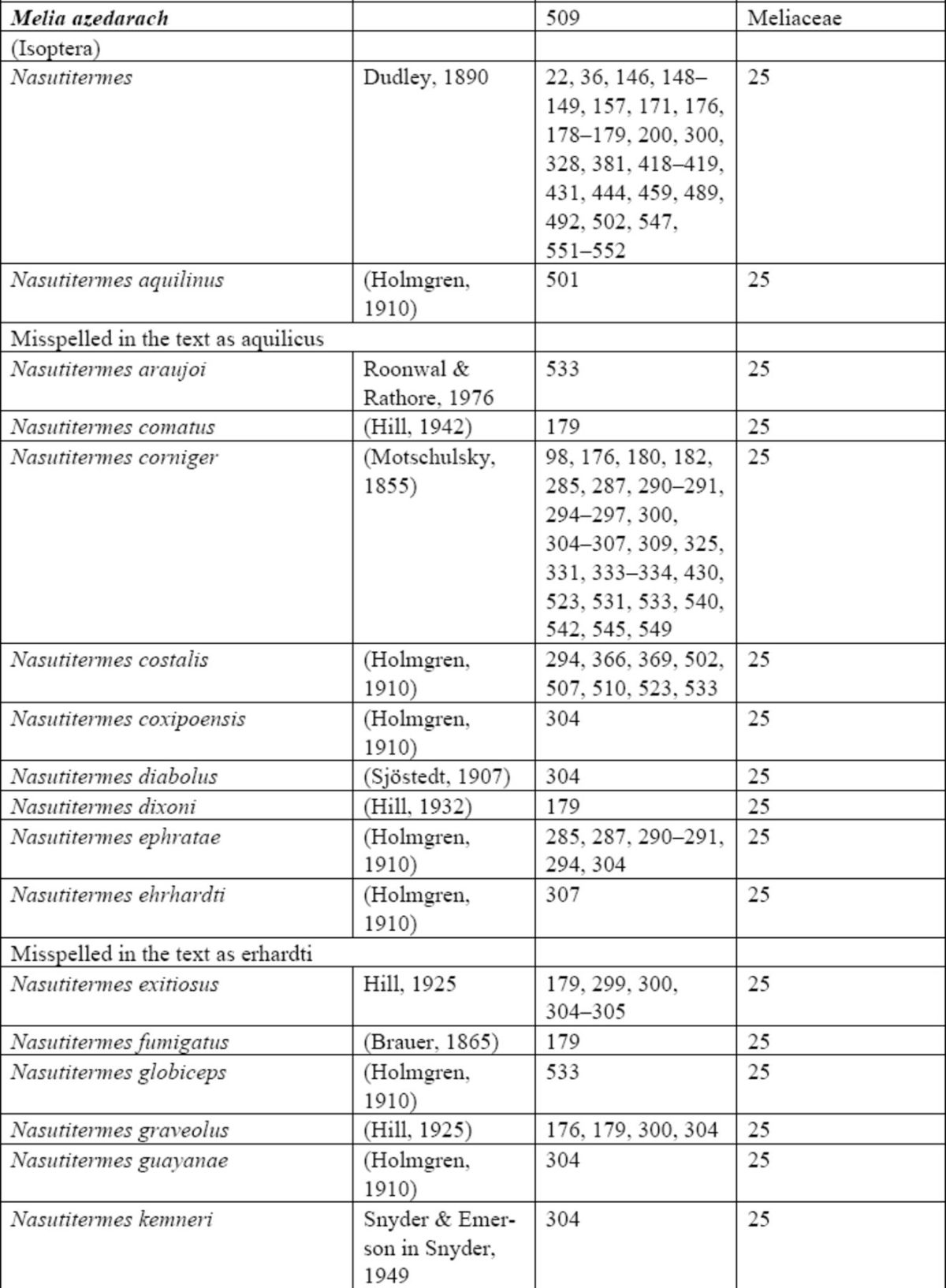

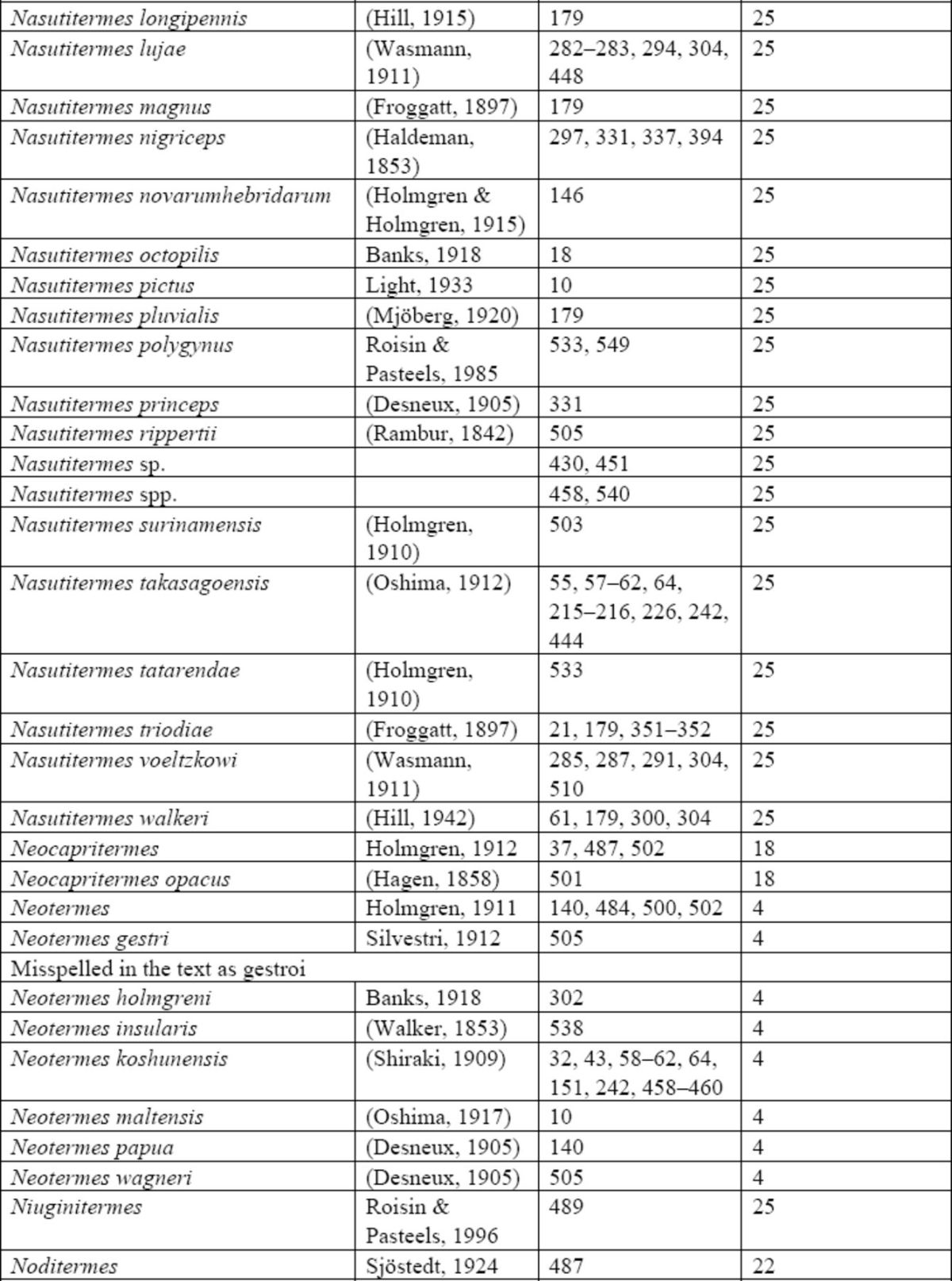

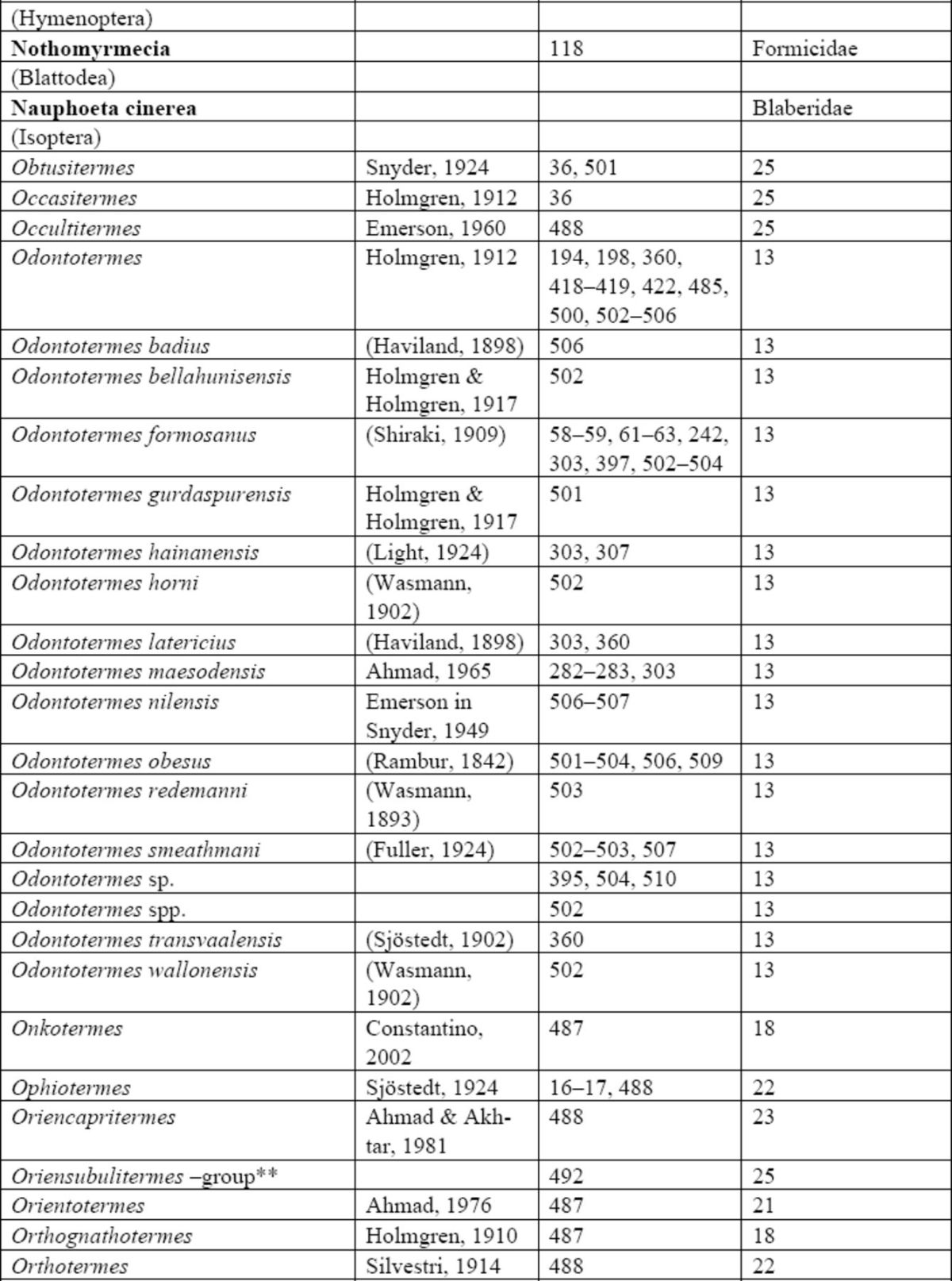

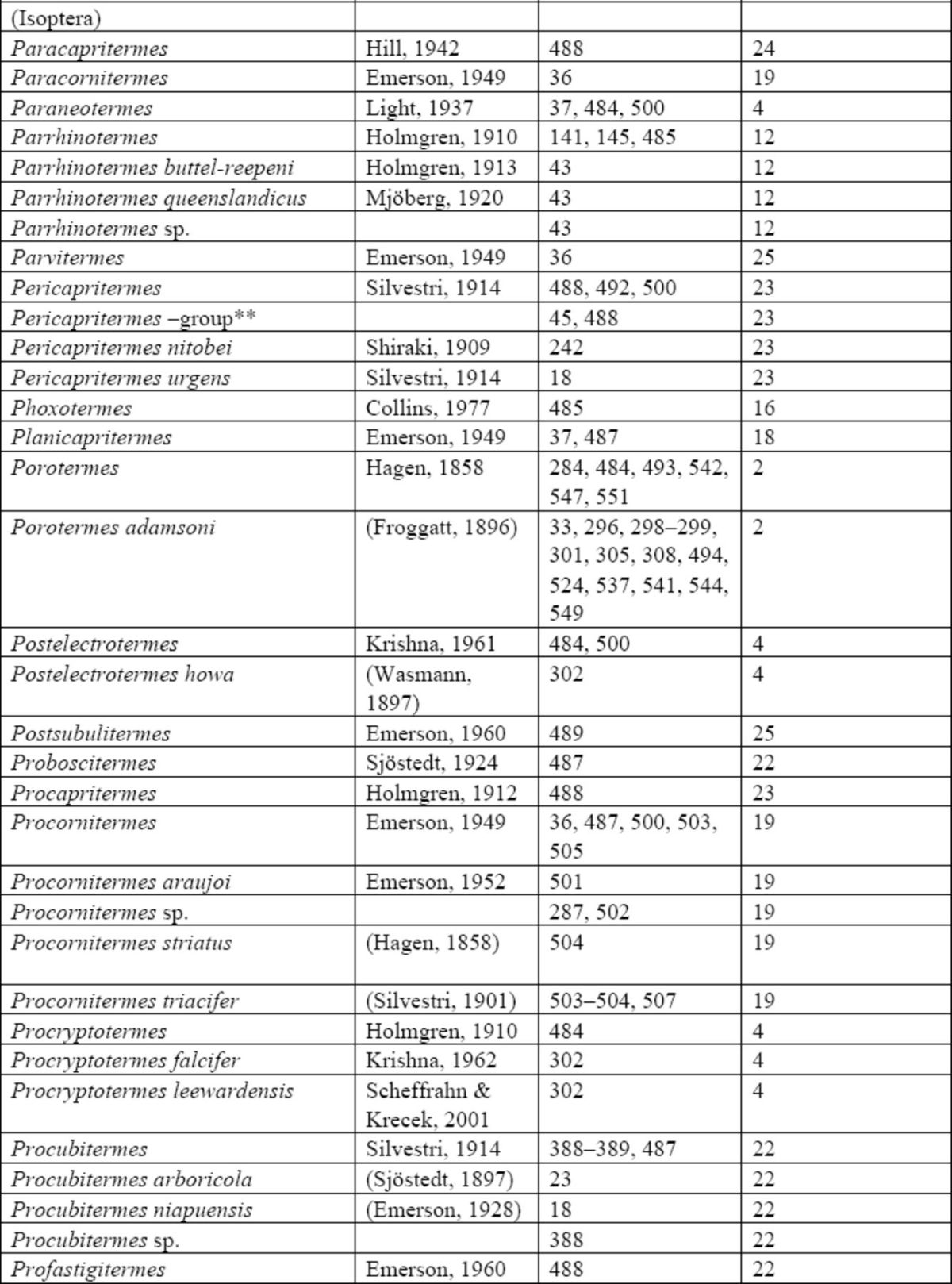

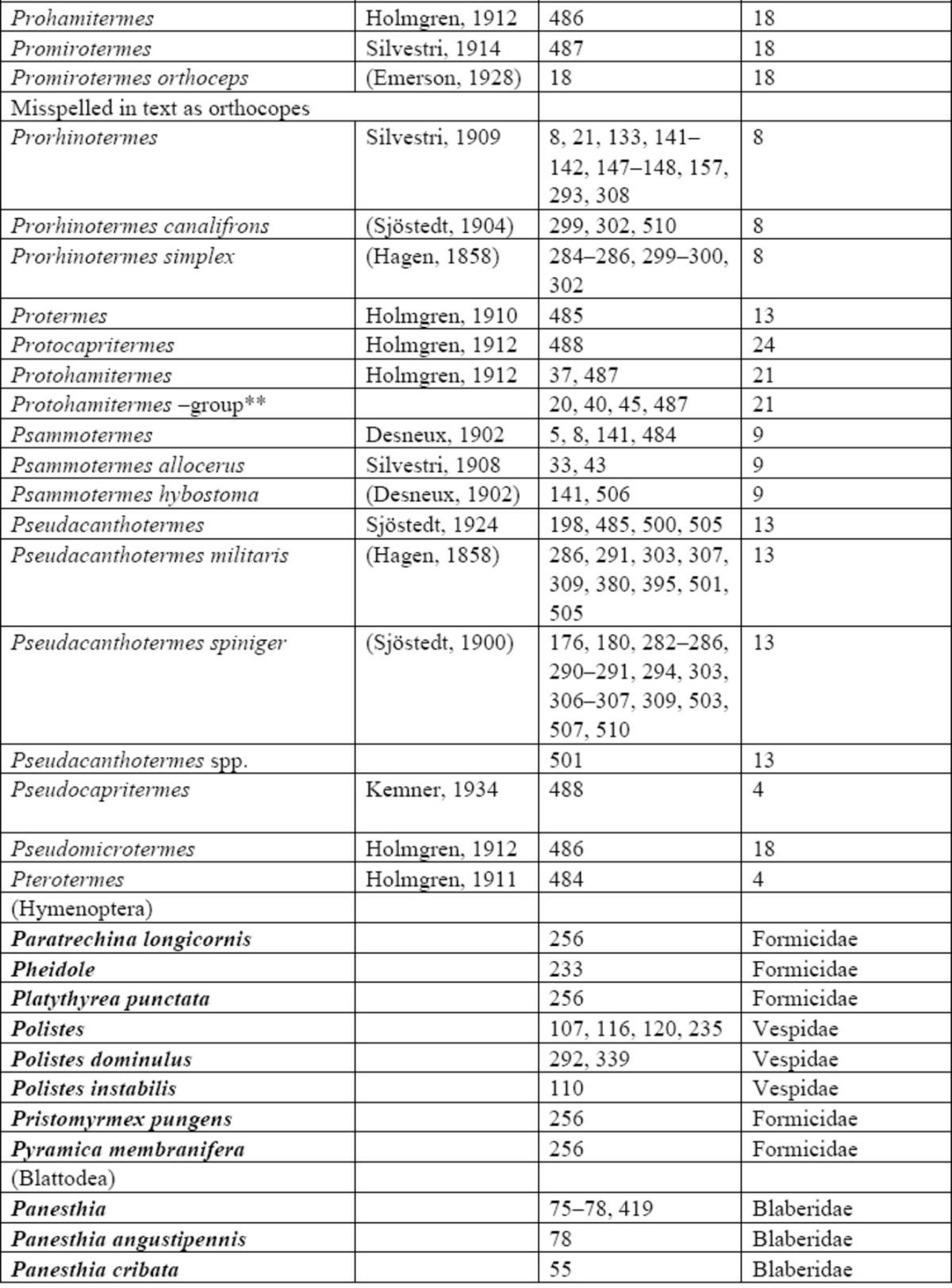

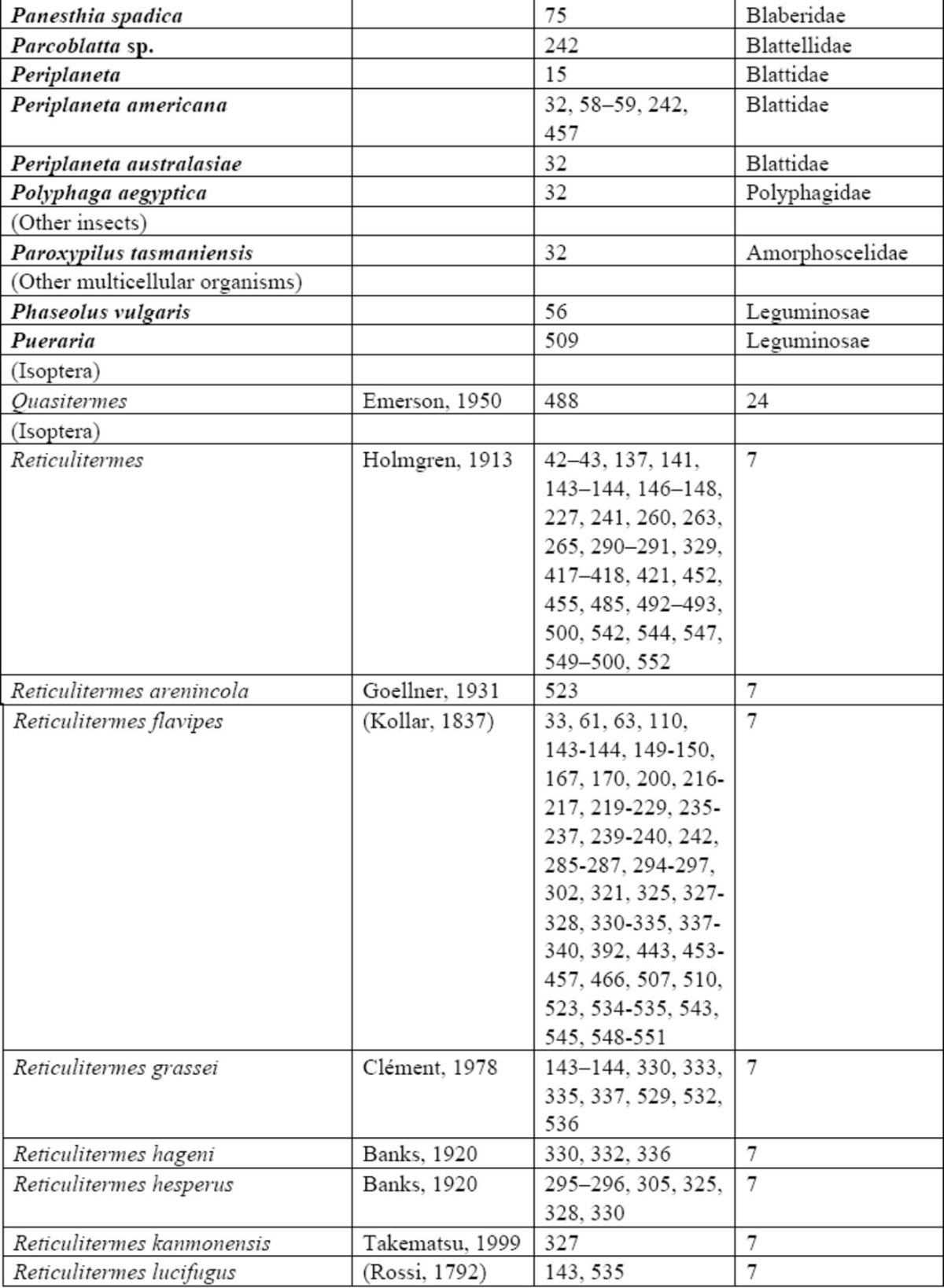

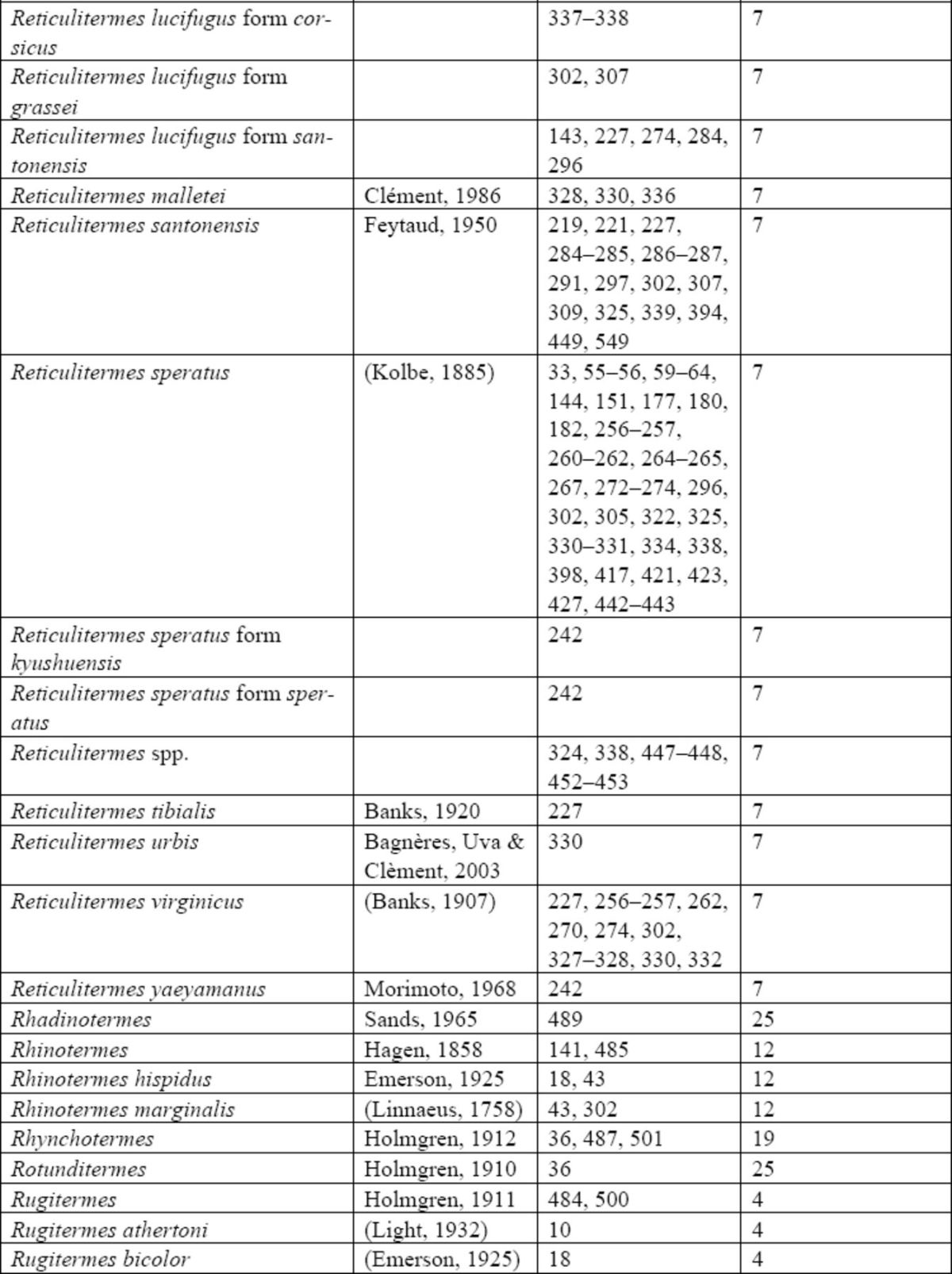

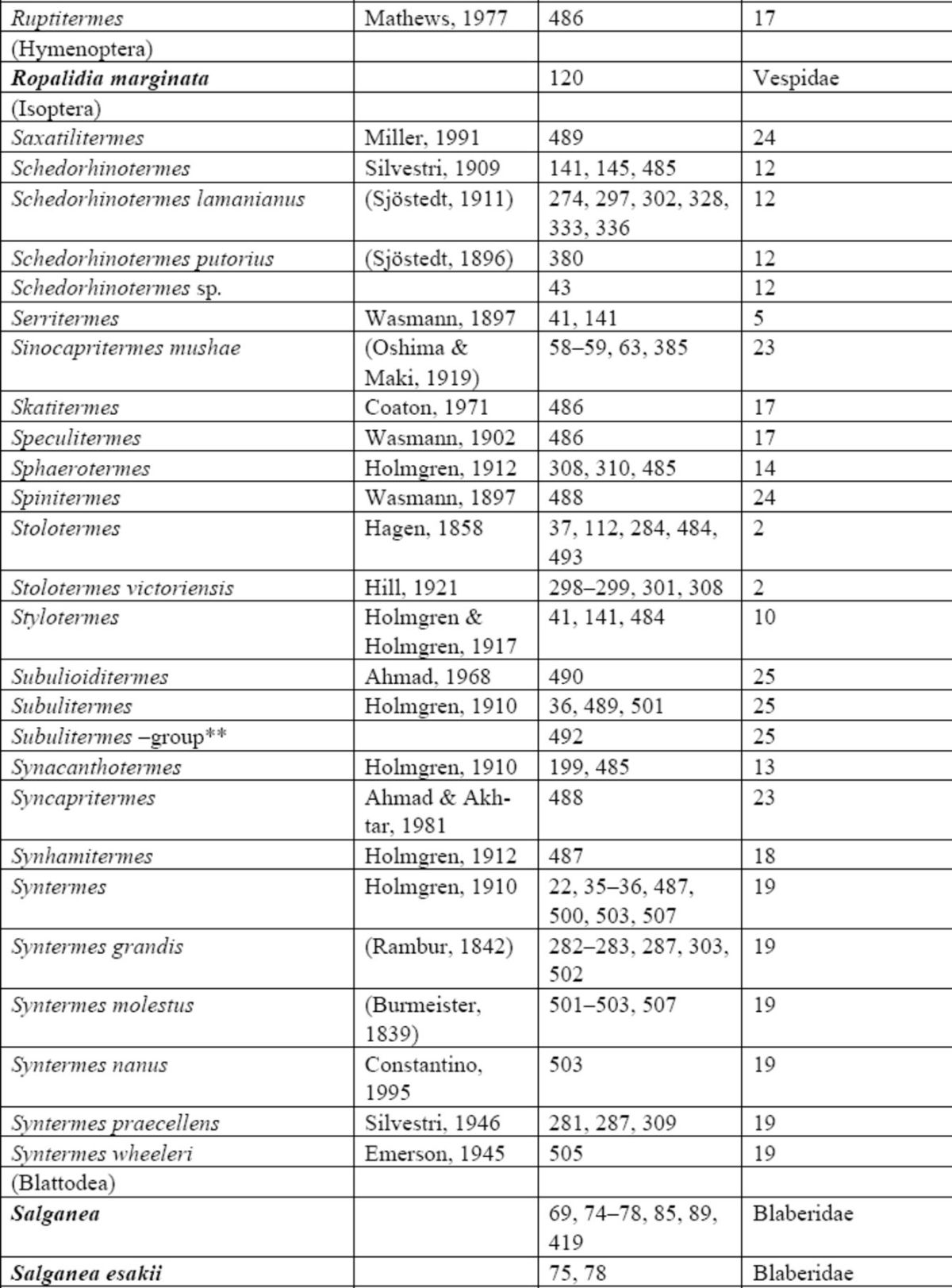

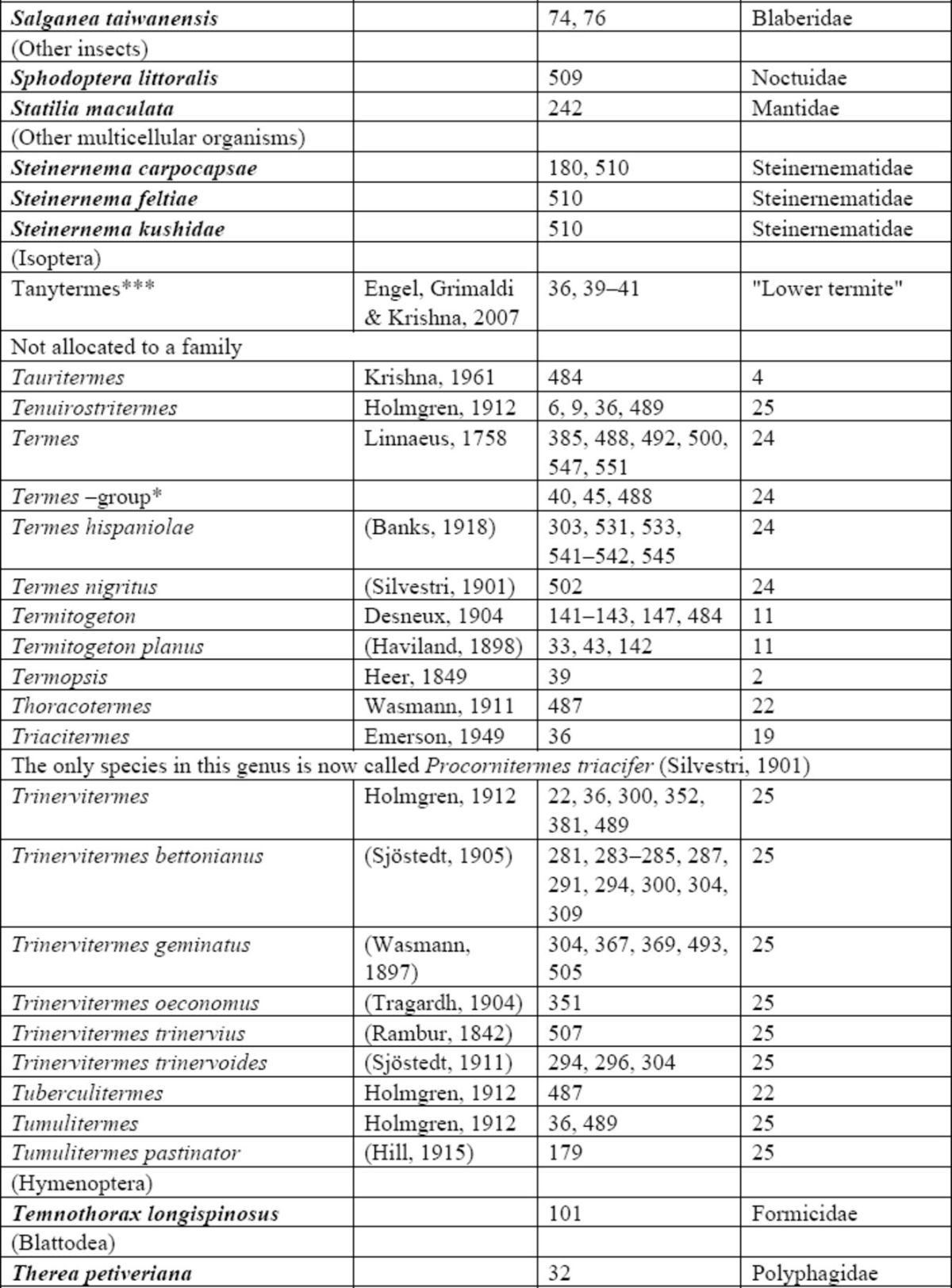

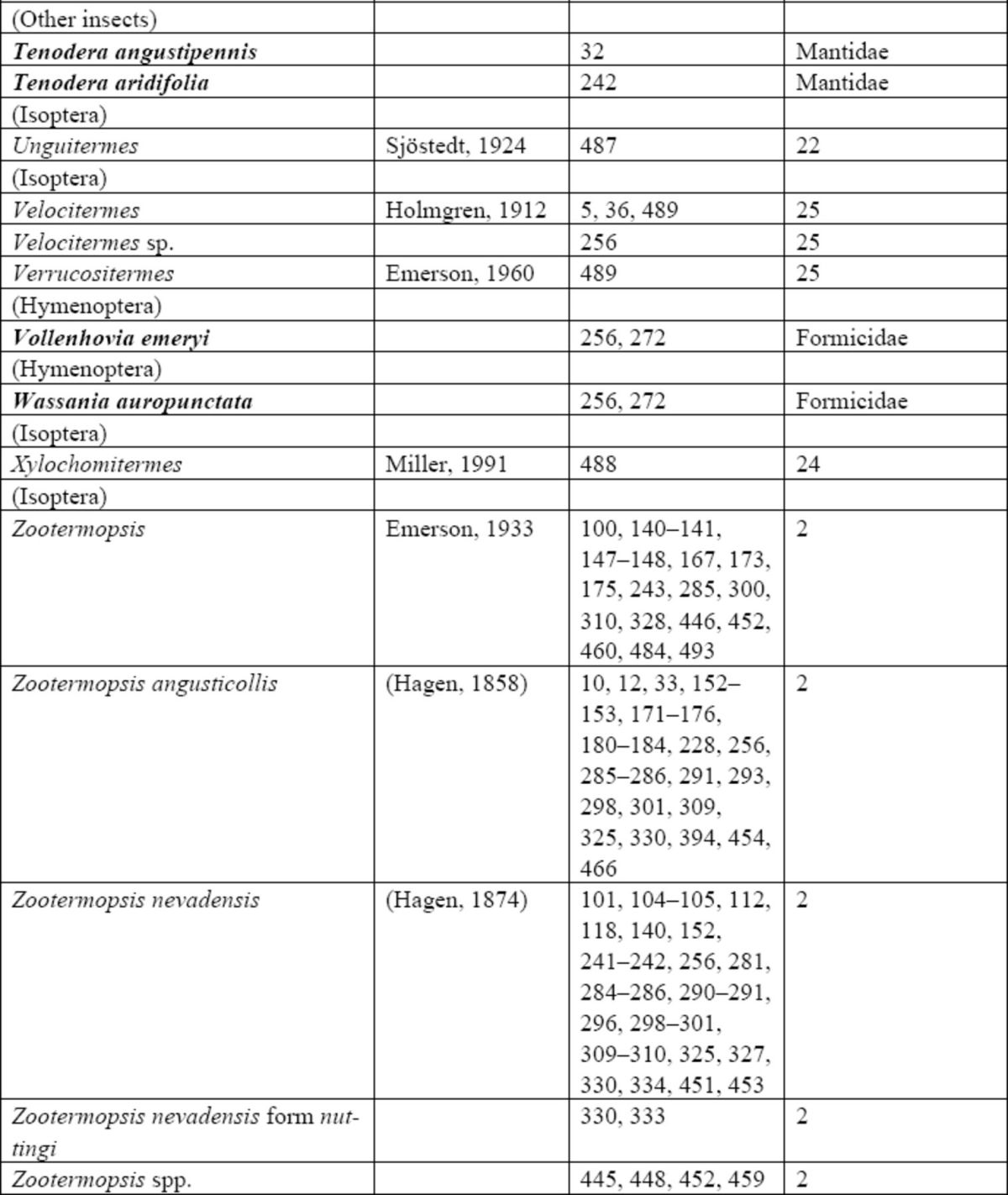
This is presented in a single table with 25 subsections, separated alphabetically. Notes deal mainly with synonyms or errors in the textbook, but some revisions and clarifications are also reported. Descriptive authorities are given for termites only. Taxonomic codes for termites are set out in Table 2 . The term “Isoptera” is informal, as the ordinal status of termites is the subject of debate ( Lo et al. 2007 ; Eggleton et al. 2007 ; Lo and Eggleton 2011 ). *this functional taxonomic group affiliation is retained from Kambhampati and Eggleton (2000) and Eggleton (2000) . **new functional taxonomic group following Davies et al. (2003) , Inward et al. (2007) , and Jones and Eggleton (2011) . *** fossil termite; for descriptive authority see Thorne et al. (2000) and Engel et al. (2009) .

Genus (refers, presumptively, to all affiliated species)

Genus sp. or spp. (refers to one or more species, not identified or possibly undescribed)

Genus and species

Genus, species, and form (geographical variant)

Genus cf. species (species identity unconfirmed)

Genus-group (refers to a FTG or presumed FTG; used with higher termites only)

Morphospecies and subspecies are excluded, though there is occasional reference to forms. Descriptive authorities are given for genus and for genus and species only. For synonymies, readers should refer to the authorities given. For fossil taxa refer to Thorne et al. (2000) and Engel et al. (2009) . In column 4, each entry is given a taxonomic code from 1 to 25. These refer to the functional taxonomic groups listed in Table 2 , and follow Kambhampati and Eggleton (2000) , Davies et al. (2003) , Inward et al. (2007) , and Jones and Eggleton (2011) . Format for the descriptive authority of termites follows convention: if a species-group taxon was described in a given genus and later transferred to another, the name of the author of the species-group name is enclosed in parentheses. Page numbers given for individual taxa do not include any chapter bibliographies. Non-termite multicellular animals are listed in bold face at the foot of each alphabetical listing, without descriptive authorities or taxonomic codes, however higher taxonomic groups (at the family level) are given and mainly follow *The Taxonomicon* ( http://taxonomicon.taxonomy.nl/ ).

For microorganisms, listings are separated by kingdoms ( Madigan and Martinko 2006 ), and therefore appear as separate tables for prokaryotes (Archaea and Eubacteria combined), fungi, and protists respectively (Tables 3–5). In each case, further affiliations at two higher taxonomic levels are given to assist in placing the symbionts within their own groups and to demonstrate the diversity of microorganisms that have been reported to be associated with termites. The microbial higher taxa should not be regarded as definitive, as there are currently no completely agreed upon classifications for any of these groups. Little work on termite virology is known to us, and only one study is cited in the book ( Ahlam et al. 1988 ).

For prokaryotes, we have classified taxa to phylum, following *The Tree of Life Web Project* ( http://www.tolweb.org ), having first identified each organism as archaeal or eubacterial. Similarly for fungi, we have allocated taxa to division (Dictyosteliomycota, Oomycota, Zygomycota, Ascomycota, and Basidiomycota), broadly following the *Tree of Life* scheme, and then to order following the *Index Fungorum* ( http://www.indexfungorum.org ). Please note that in these schemes, the groups “Hyphomycetes,” “Deuteromycetes,” and “Fungi Imperfecti” are all subsumed under Ascomycota. No yeasts are mentioned in the book. For protists, we have identified taxa at class level (most are either oxymonads or parabasalians) and then to order or family as recommended by The Taxonomicon ( http:///www.taxonomy.nl/taxonomicon ), and following recent reviews by Brugerolle and Radek (2006) and Ohkuma and Brune (2011; chapter 15 of the book). Amoeboid grade protists occur in some termites, but no taxonomic names are reported in the book.

### Index of termites and other animals, excluding protists

**Table 2:**
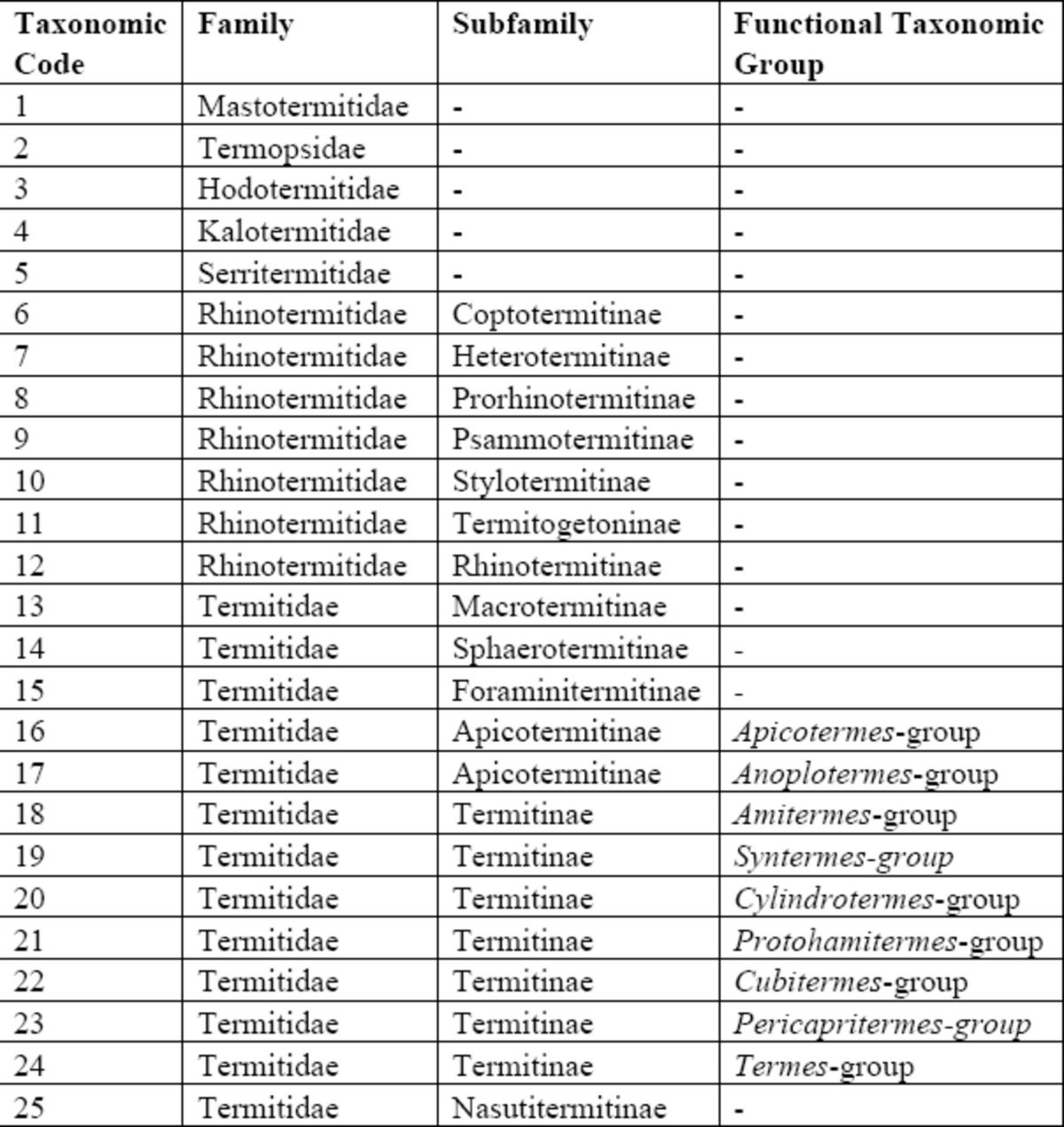
Key to taxonomic codes. The codes abbreviate the affiliation of each termite listed in the alphabetical index (this paper), following the scheme developed by Davies et al. (2003) and Inward et al. (2007b) and used in Table 17.2 Chapter 17 ( *Global Biogeography of Termites: a Compilation of Sources* , pp. 477–498)

### Index of microorganisms

**Table 3:**
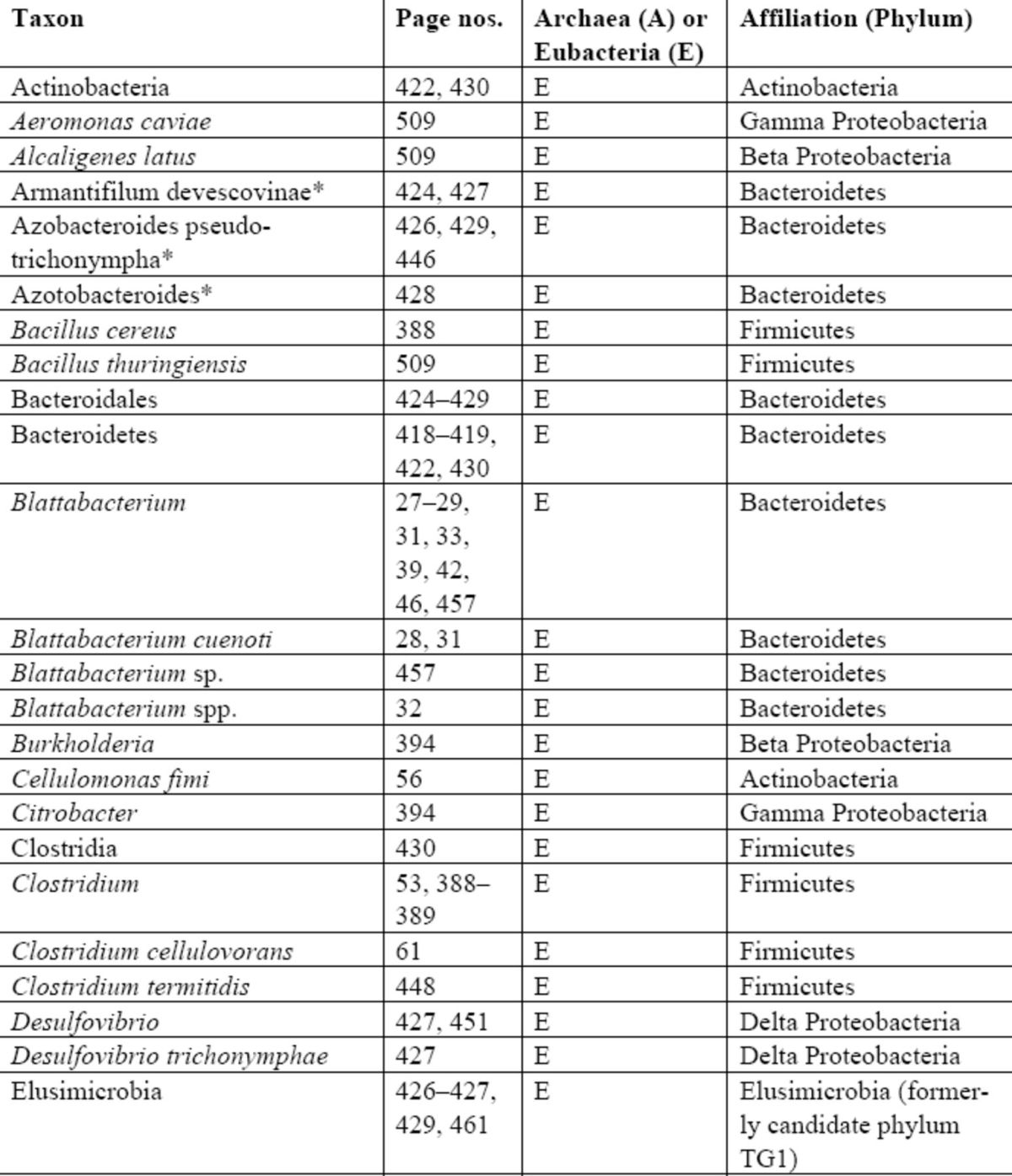

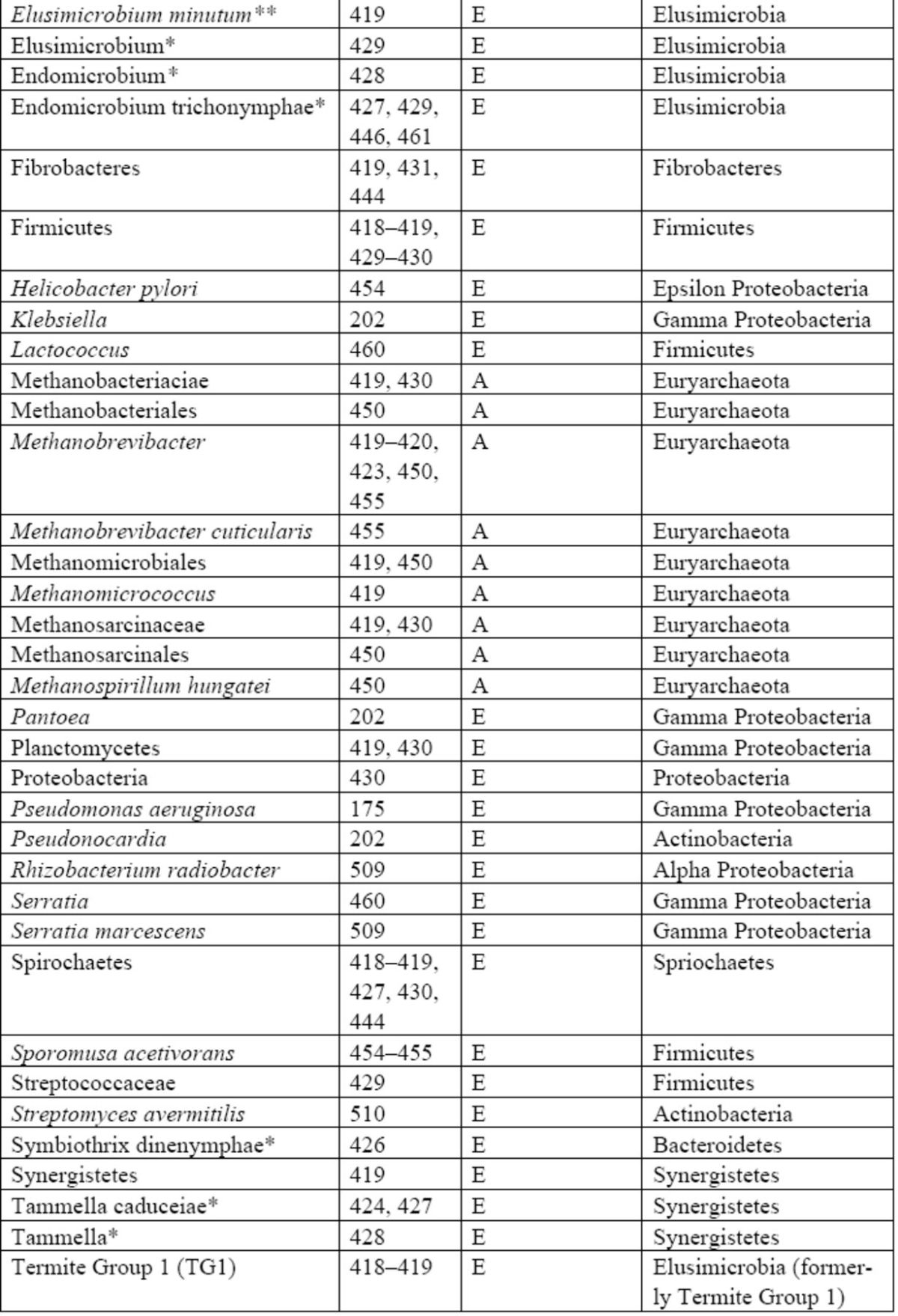

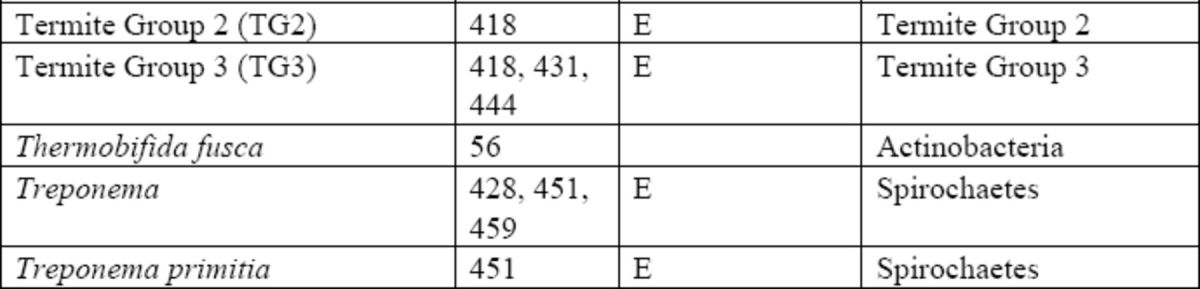
Index of prokaryotes. Taxa with an asterisk (*) have *Candidatus* status (characterised but uncultivable). One strain of the genus *Elusimicrobium* *, *E. minutum* (**) has been cultured ( Geissinger et al. 2009 ). Higher classifications based on the *Tree of Life* web project for Eubacteria ( http://tolweb.org/Eubacteria ) and Archaea ( http://tolweb.org/Archaea/4 ), but are not intended to be definitive. No taxonomic notes are given.

PROKAROTES

**Table 4:**
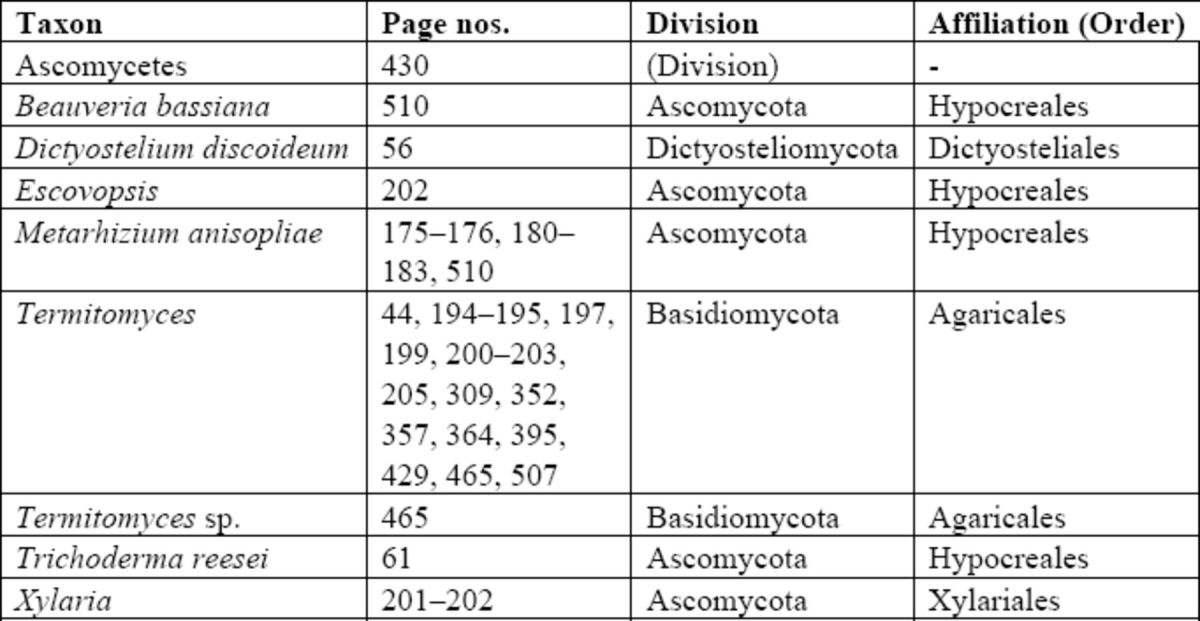
Index of fungi. This is presented as a single table without subdivision, ordered alphabetically. Classifications follow the same scheme as Bignell and Jones (2009) , based on the *Index Fungorum* ( http://www.indexfungorum.org/names/names.asp ), but are not intended to be definitive. No taxonomic notes are given.

PROTISTS

**Table 5:**
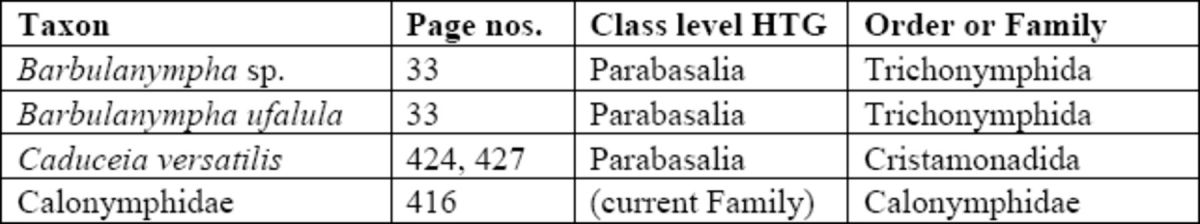

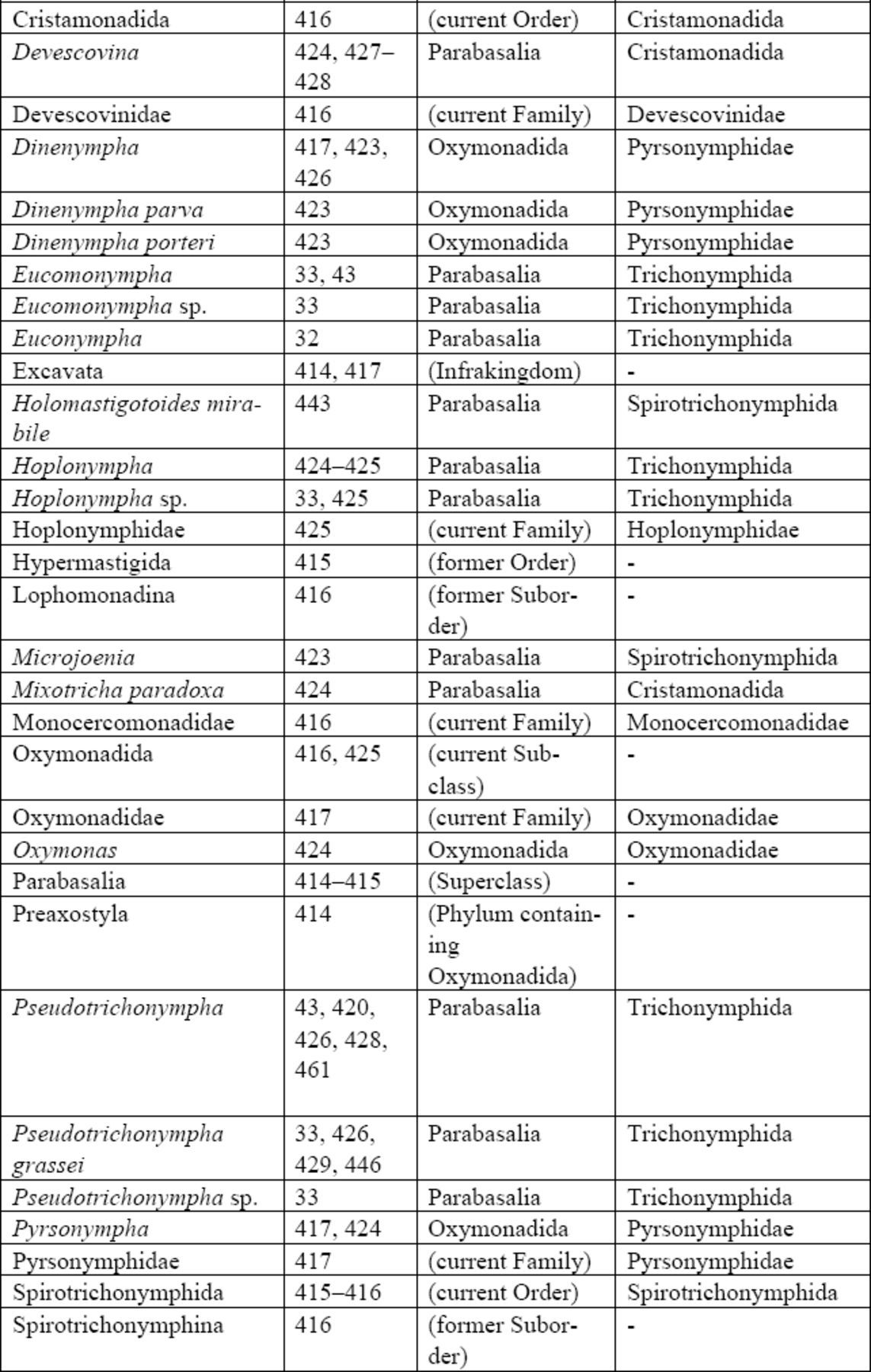

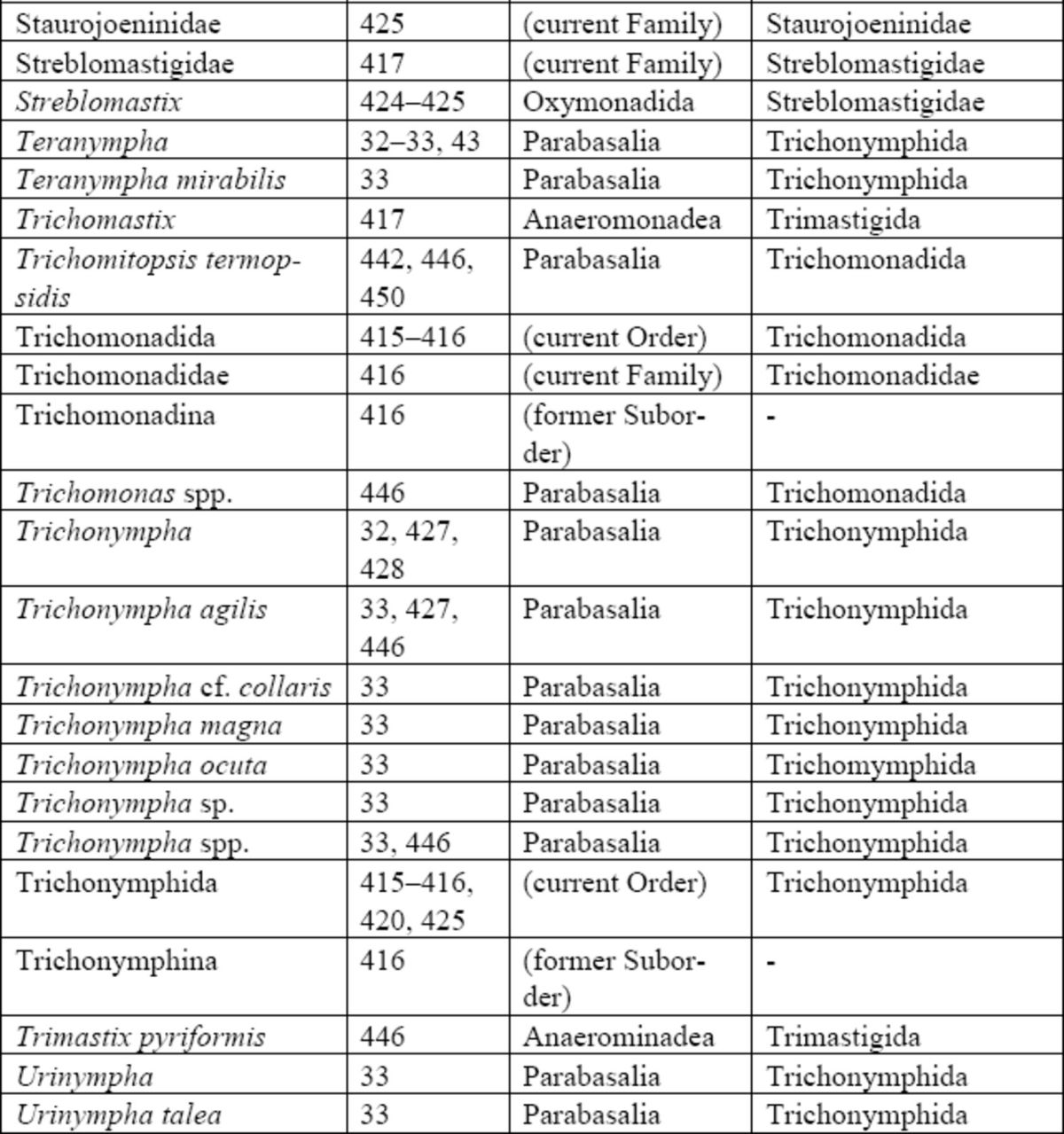
Index of protists. This is presented in a single table without subdivision, ordered alphabetically. No taxonomic notes are given. Classifications follow Brugerolle and Radek (2006) , Ohkuma and Brune (2011) , and *The Taxonomicon* ( http://taxonomicon.taxonomy.nl/Default.aspx ), but are not intended to be definitive.

## Abbreviations

FTG: functional taxonomic group
HTG: higher taxonomic group
